# Supplementary material for: Loss of the lysosomal protein CLN3 triggers c-Abl-dependent YAP1 pro-apoptotic signaling
Source: EMBO Rep. 2025 Nov 6;26(24):6096–120. doi: 10.1038/s44319-025-00613-3 (PMC12714701; doi:10.1038/s44319-025-00613-3)
Supplement: Supplementary file 1 — Table EV1 [file 44319_2025_613_MOESM1_ESM.pdf]

Table EV1 – List of human primers and siRNA sequences.

Primers	*Sequences (5'-3')*
**h_PUMA_F**	ATCAATCCCATTGCATAGGTTTAG
**h_PUMA_R**	ACTAAGGCTGGGGCGCTTC
**h_TP53AIP1_F**	GGCTCAGACACACACACCT
**h_TP53AIP1_R**	GGCCTGTCTCTAAGCACTGT
**h_Bax_F**	ATGTTTTTCTGACGGCAACTTC
**h_Bax_R**	ATCAGTTCCGGCAACCTTG
**h_TP73_F**	CCCACCACTTTGAGGTCACT
**h_TP73_R**	GGCGATCTGGCAGTAGAGTT
**h_DR5_F**	GTGATTCAGGTGAAGTGGAGC
**h_DR5_R**	CGACCTTGACCATCCCTCTG
**hYAP1_F**	TAGCCCTGCGTAGCCAGTTA
**hYAP1_R**	TCATGCTTAGTCCACTGTCTGT
**S16_F**	AGGAGCGATTTGCTGGTGTGG
**S16_R**	GCTACCAGGGCCTTTGAGATG
**Cycloph_F**	GGCAAATGCTGGACCAAACACAA
**Cycloph_F**	GGCAAATGCTGGACCAAACACAA
**Cycloph_R**	GTAAAATGCCCGCAAGTCAAAAG
**mYAP1_F**	CGGCAGTCCTCCTTTGAGAT
**mYAP1_R**	GGTCCTGCCATGTTGTTGTC
**mTP53_F**	AACTATGGCTTCCACCTGGG
**mTP53_R**	TGAGGGGAGGAGAGTACGTG
**mBBC3_F**	GGATGGCGGACGACCTCAA
**mBBC3_R**	TCGGTGTCGATGCTGCTCTT
**mBAX_F**	ATCCAAGACCAGGGTGGCTG
**mBAX_R**	TCACTGTCTGCCATGTGGGG
**mTrp73_F**	TCACCTTCCAGCAGTCGAGC
**mTrp73_R**	TGGATGGGGCATGTCTTAGCA
**mDr5_F**	AGCCCATCAAGAGGACCCTG
**mDr5_R**	AGGCTTGCAGTTCCCTTCTGA
**mcd68_F**	TCAGCTGCCTGACAAGGGAC
**mcd68_R**	GCAGCAAGAGGGACTGGTCA
**mCln3AC_F**	CCTCCAGGAAAGGTGGACAG
**mCln3AC_R**	GCTCGAAAAGTCCCTGGTTG

siRNA	***Sequences (5'-3')***
**h_siCLN3_1**	UUGUUCUUUCAAGGUCUAUUCUUUAUAGACCUUGAA
**h_siCLN3_2**	GCAGUACCGAUGGUACCAUAGCAUCUGGUACCAUCG
**h_siYAP_1**	ACGGUAGAUAUUACUGACAAUUCAUCAGAUAAUAU
**h_siYAP_2**	GCUGCCACCAAGCUAGAUUUUCUUUAUCUAGCUUGG
**h_siPLA2G15_1**	GUAUCUGGAUUCUGGCAAACUUUUAUUGCCAGAAUC
**h_SiPLA2G15_2**	AGACCGAAAGCUACUUCACAGAUUGUGAAGUAGCUU
**m_siCLN3**	GCAGUACCGAUGGUACCA
