# Supplementary figures and images for: Loss of the lysosomal protein CLN3 triggers c-Abl-dependent YAP1 pro-apoptotic signaling
Source: EMBO Rep. 2025 Nov 6;26(24):6096–120. doi: 10.1038/s44319-025-00613-3 (PMC12714701; doi:10.1038/s44319-025-00613-3)

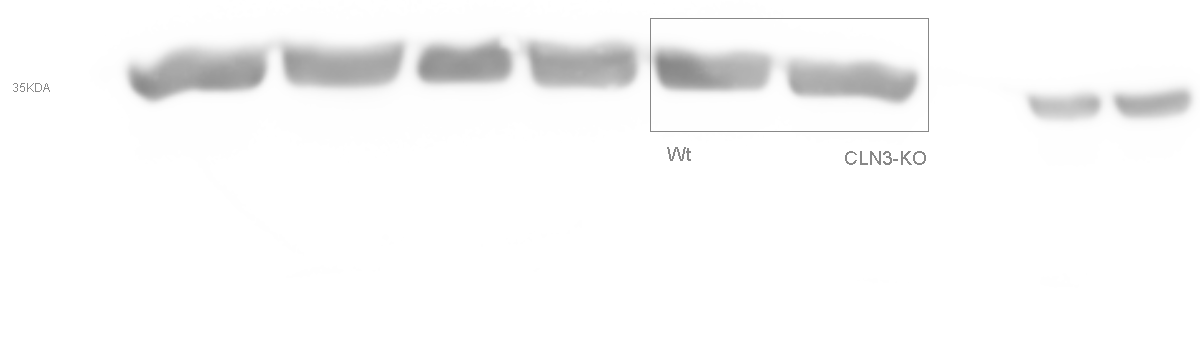

Supplement: Supplementary file 4 — Source data Fig. 2 [file 44319_2025_613_MOESM4_ESM.zip › Figure 2/A/ARPE GAPDH.tif]

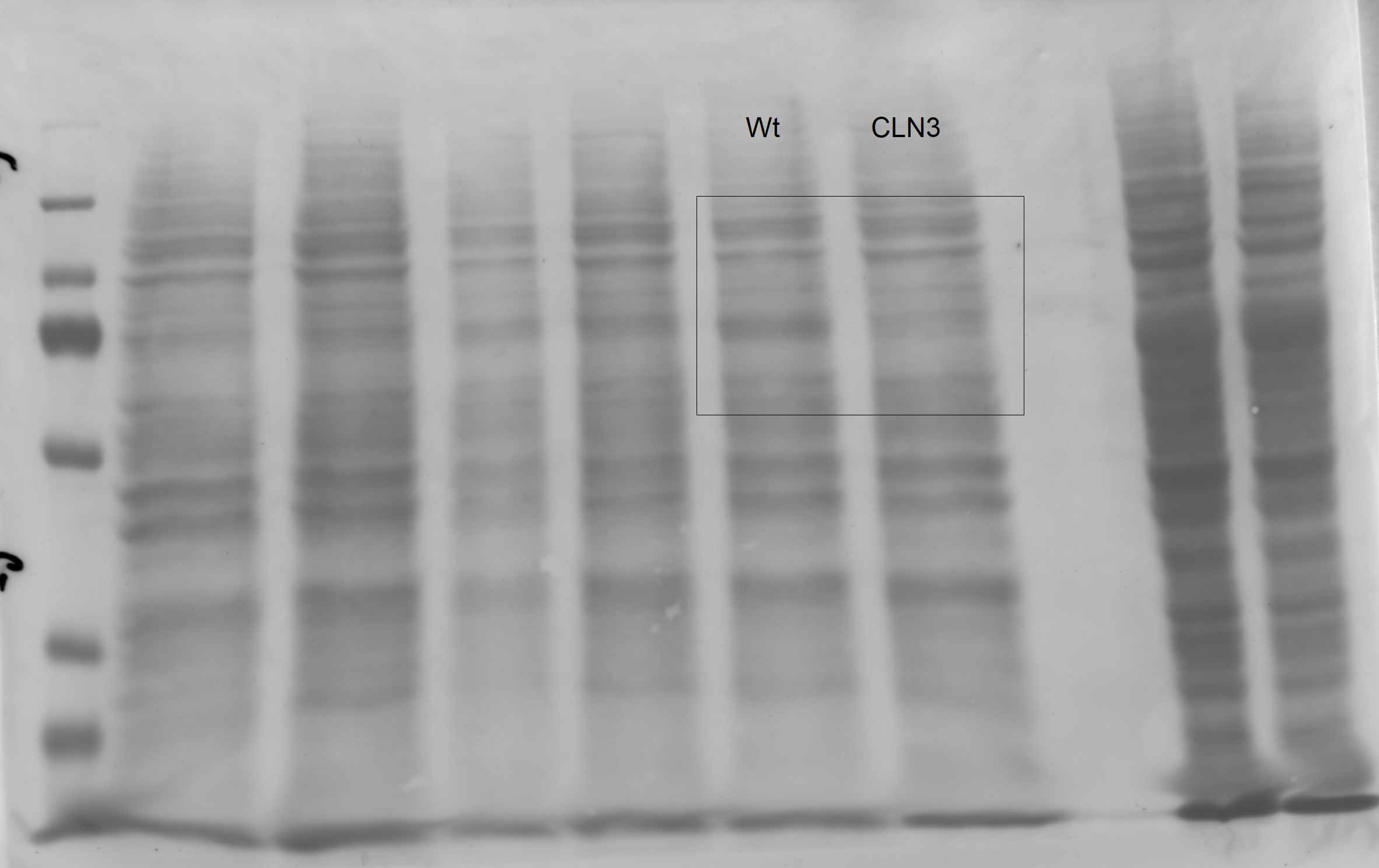

Supplement: Supplementary file 4 — Source data Fig. 2 [file 44319_2025_613_MOESM4_ESM.zip › Figure 2/A/ARPE ponceau.tif]

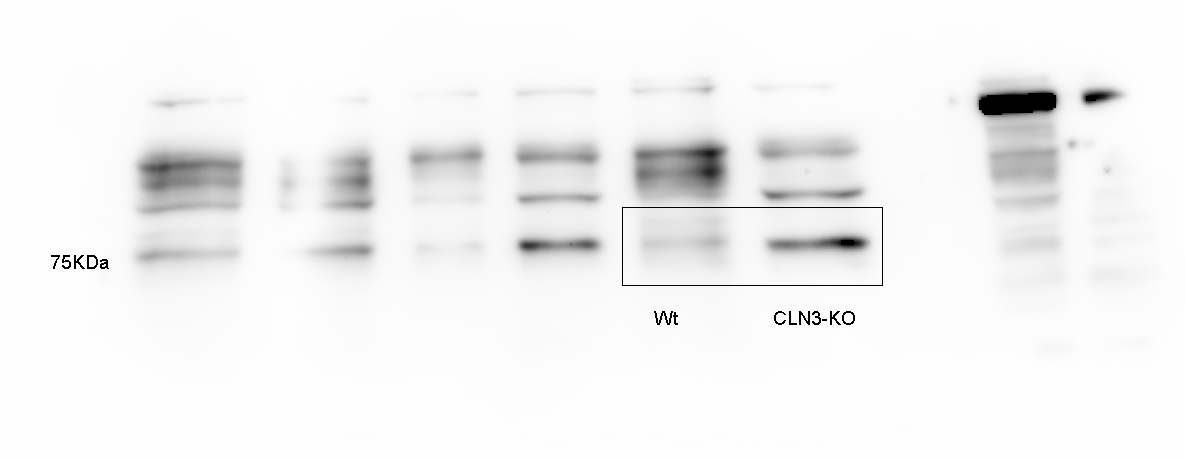

Supplement: Supplementary file 4 — Source data Fig. 2 [file 44319_2025_613_MOESM4_ESM.zip › Figure 2/A/ARPE pYAP y357.tif]

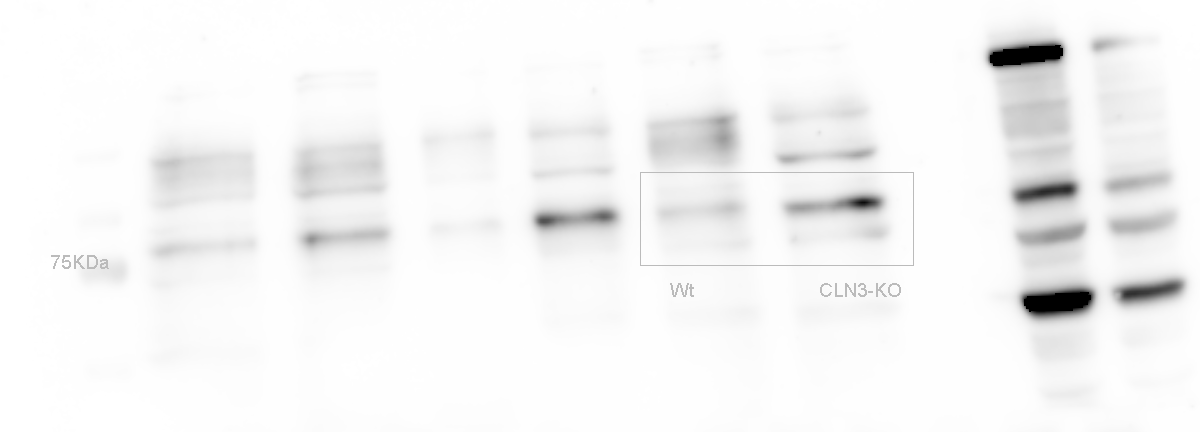

Supplement: Supplementary file 4 — Source data Fig. 2 [file 44319_2025_613_MOESM4_ESM.zip › Figure 2/A/ARPE YAP total.tif]

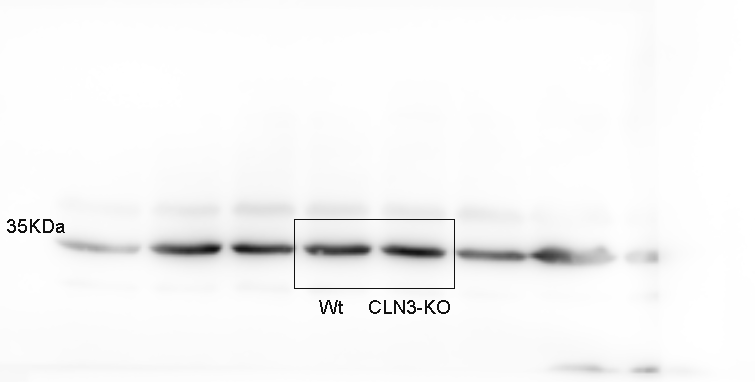

Supplement: Supplementary file 4 — Source data Fig. 2 [file 44319_2025_613_MOESM4_ESM.zip › Figure 2/A/HEK GAPDH.tif]

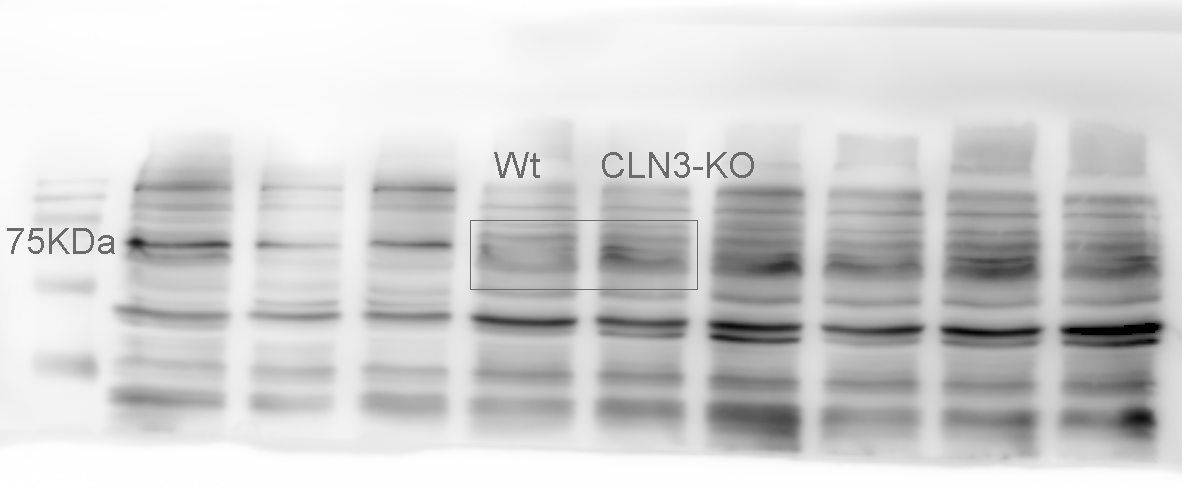

Supplement: Supplementary file 4 — Source data Fig. 2 [file 44319_2025_613_MOESM4_ESM.zip › Figure 2/A/HEK pYAP y357.tif]

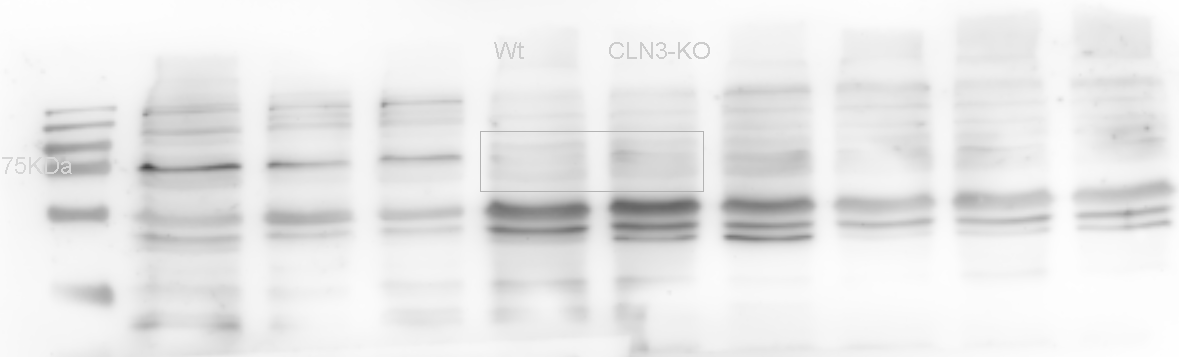

Supplement: Supplementary file 4 — Source data Fig. 2 [file 44319_2025_613_MOESM4_ESM.zip › Figure 2/A/HEK Yap total.tif]

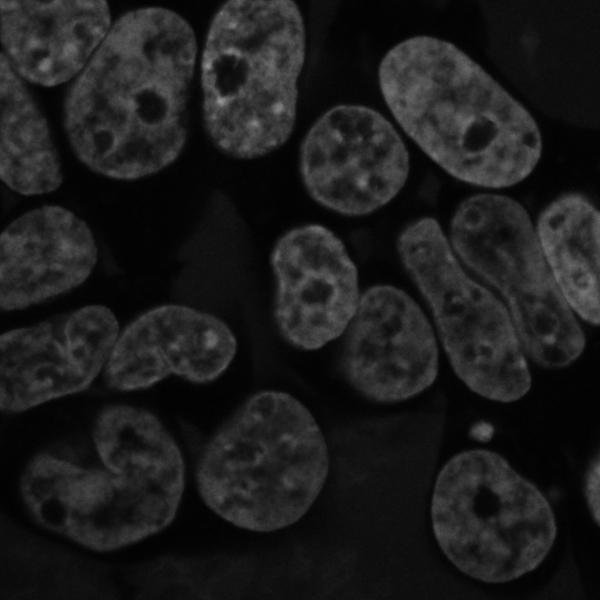

Supplement: Supplementary file 4 — Source data Fig. 2 [file 44319_2025_613_MOESM4_ESM.zip › Figure 2/B/KO CLN3_ pT357-YAP 647 image 1-2.tif]

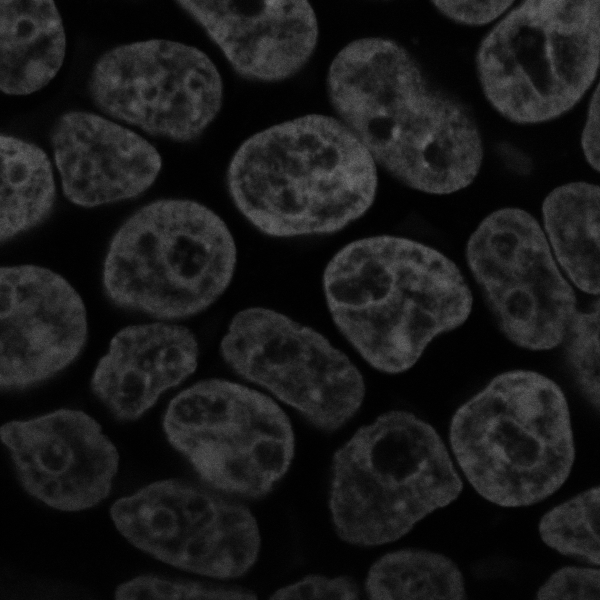

Supplement: Supplementary file 4 — Source data Fig. 2 [file 44319_2025_613_MOESM4_ESM.zip › Figure 2/B/Wt pT357-YAP 647 image 4-1.tif]

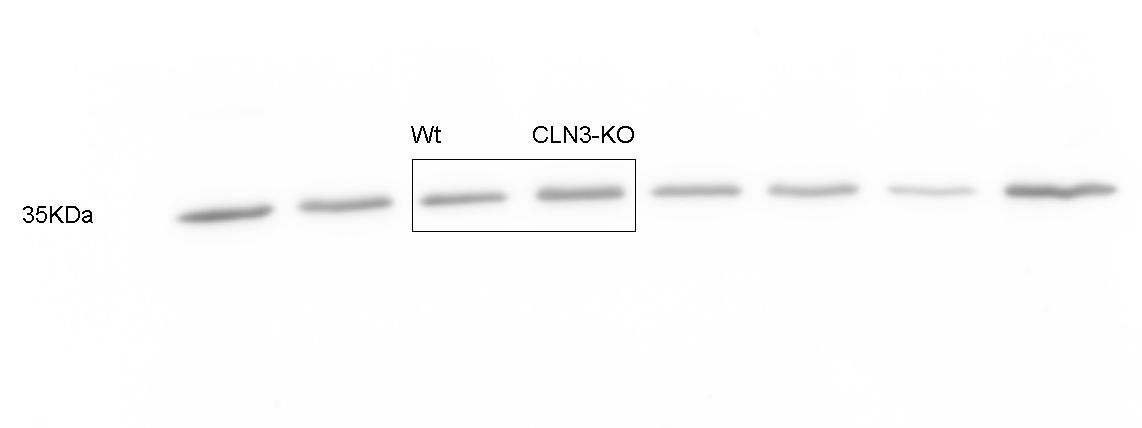

Supplement: Supplementary file 4 — Source data Fig. 2 [file 44319_2025_613_MOESM4_ESM.zip › Figure 2/C/2C GAPDH.tif]

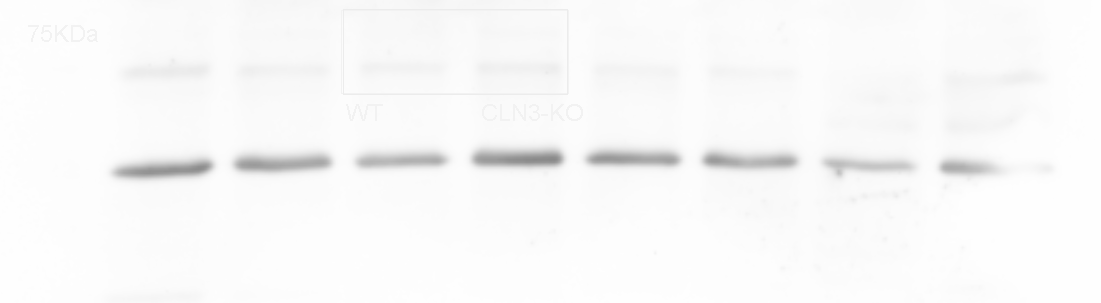

Supplement: Supplementary file 4 — Source data Fig. 2 [file 44319_2025_613_MOESM4_ESM.zip › Figure 2/C/2C HEK Yap total.tif]

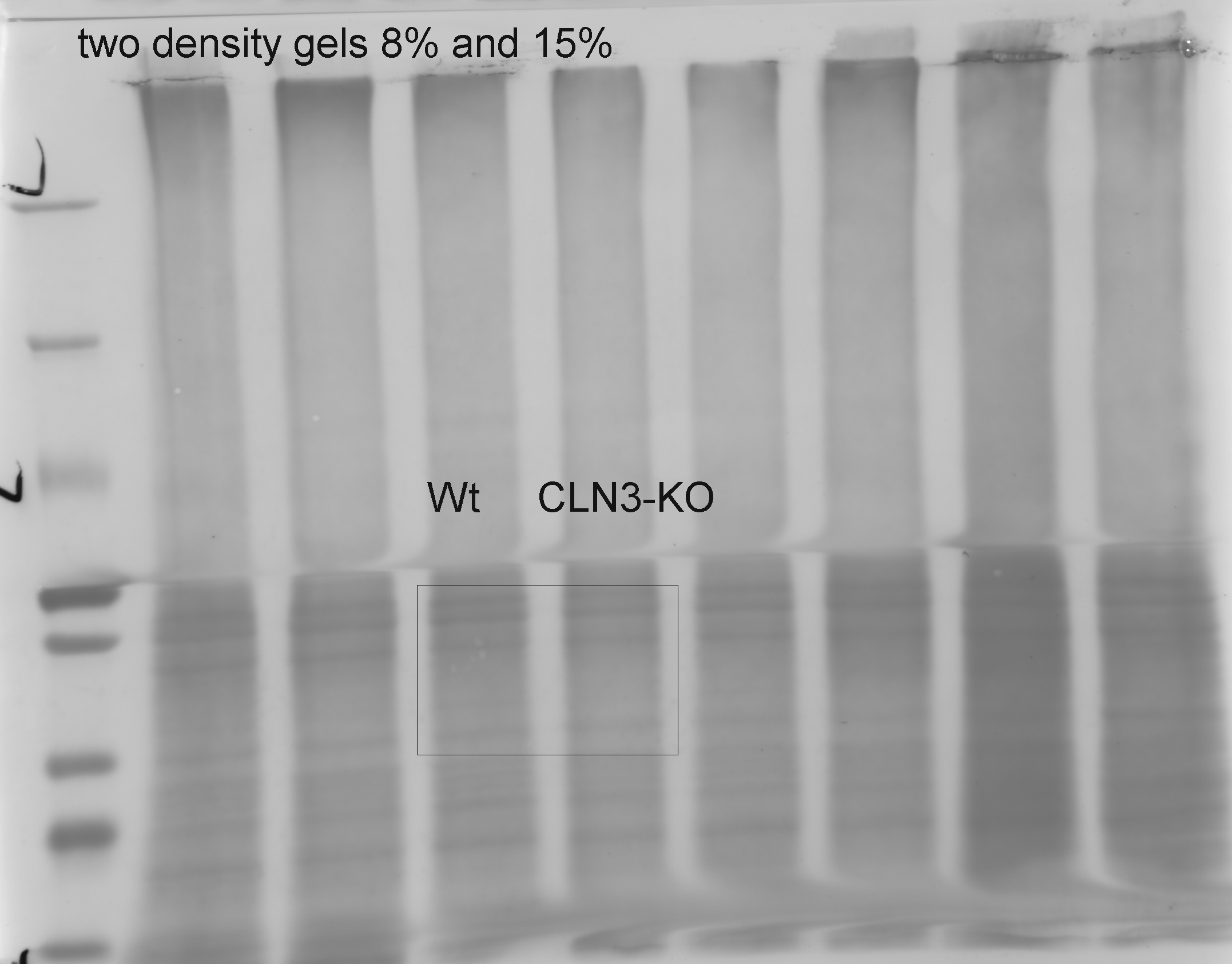

Supplement: Supplementary file 4 — Source data Fig. 2 [file 44319_2025_613_MOESM4_ESM.zip › Figure 2/C/2C ponceau.tif]

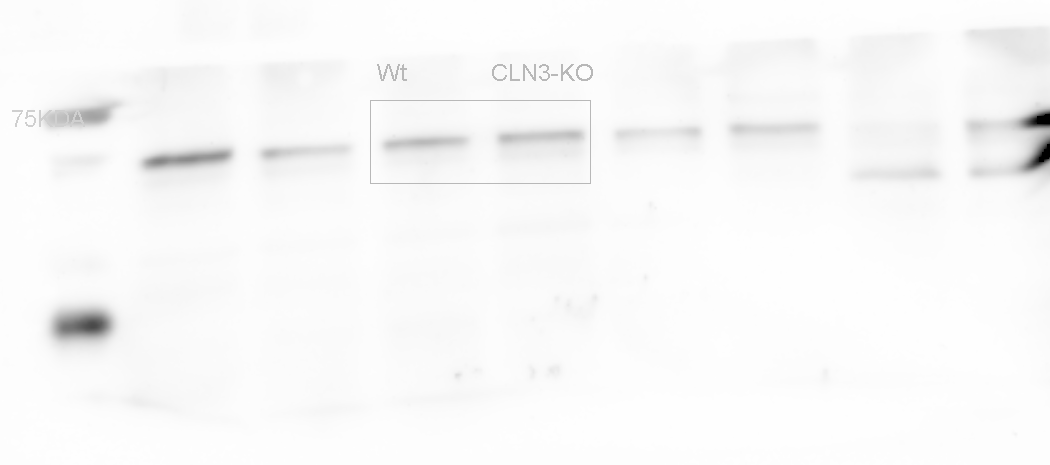

Supplement: Supplementary file 4 — Source data Fig. 2 [file 44319_2025_613_MOESM4_ESM.zip › Figure 2/C/2C pYap S127.tif]

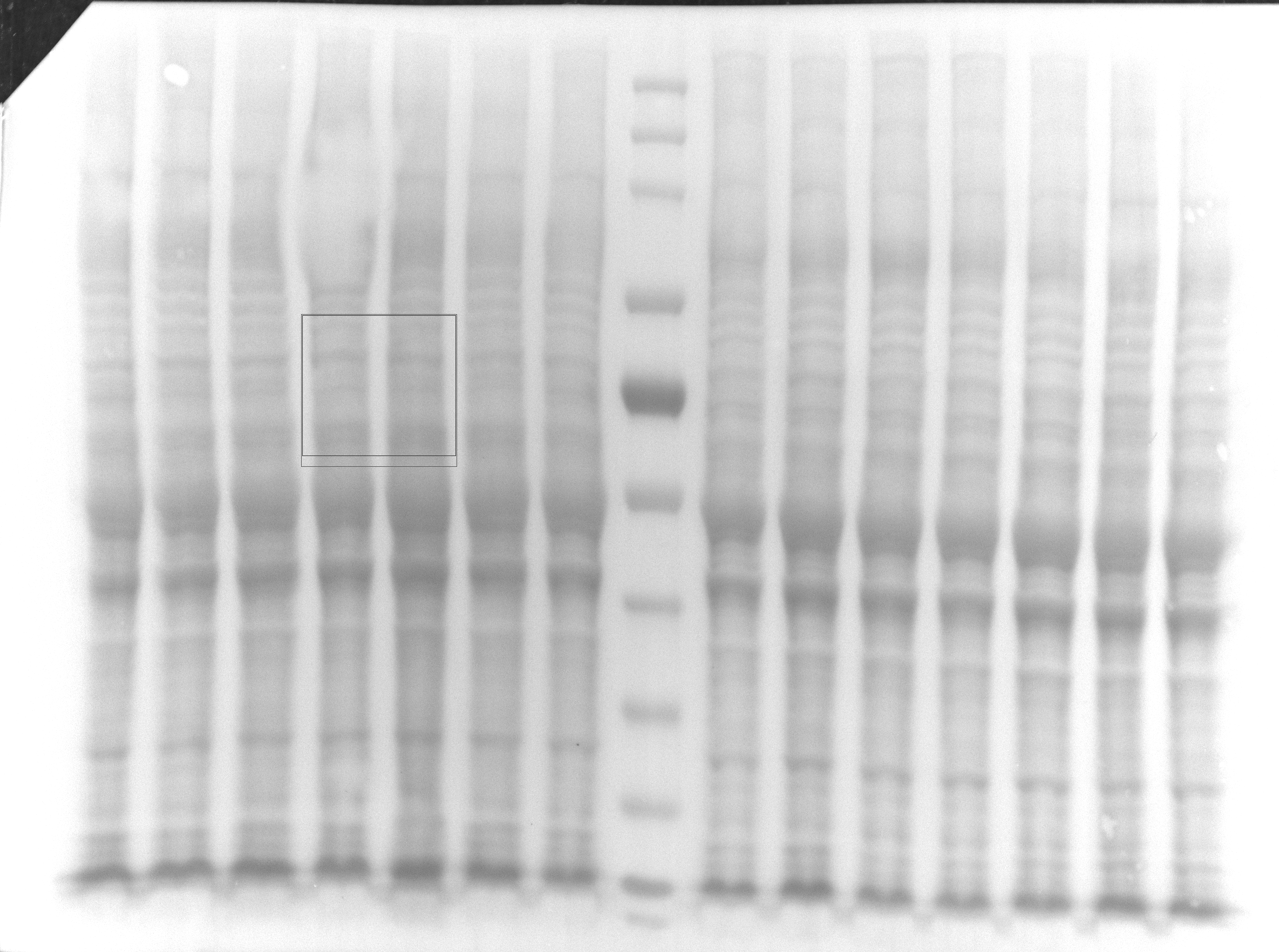

Supplement: Supplementary file 4 — Source data Fig. 2 [file 44319_2025_613_MOESM4_ESM.zip › Figure 2/E/2E ponceau.tif]

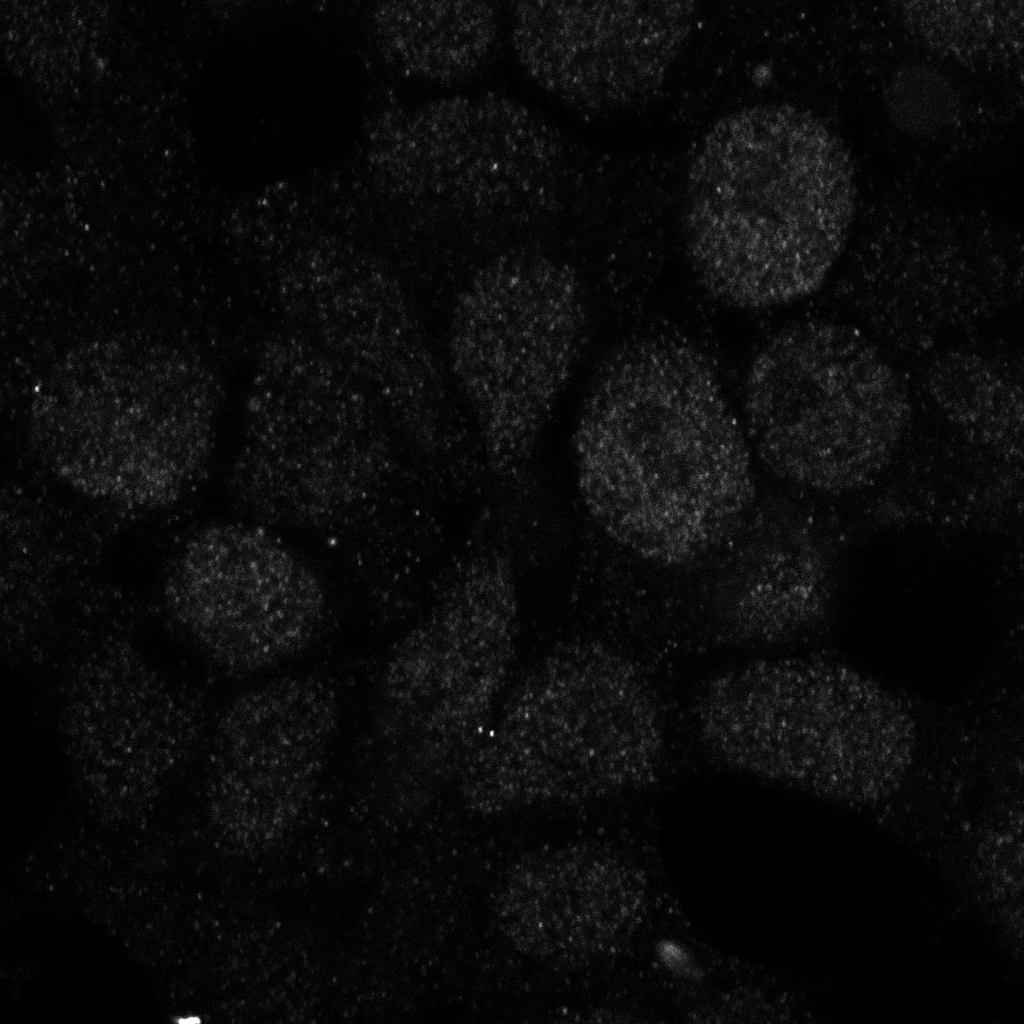

Supplement: Supplementary file 4 — Source data Fig. 2 [file 44319_2025_613_MOESM4_ESM.zip › Figure 2/F/CLN3-KO p73.tif]

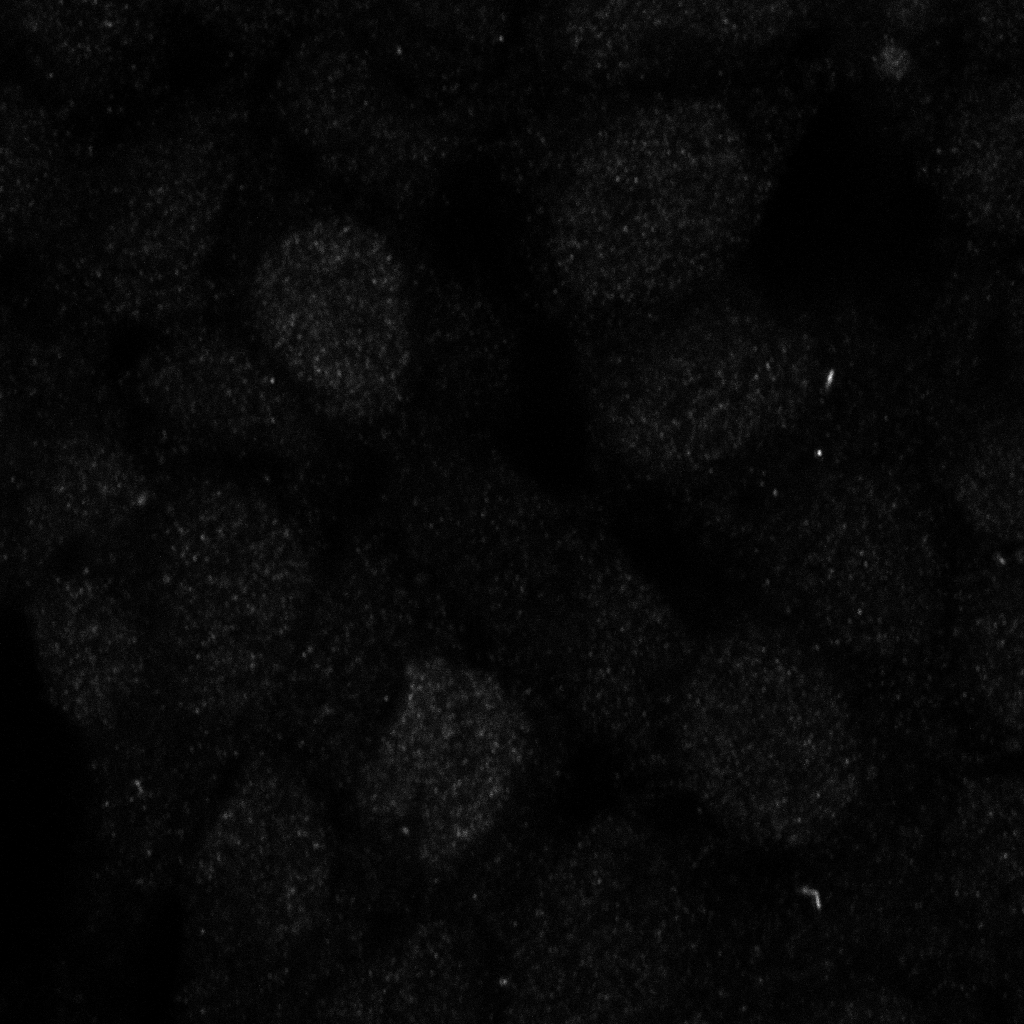

Supplement: Supplementary file 4 — Source data Fig. 2 [file 44319_2025_613_MOESM4_ESM.zip › Figure 2/F/WT p73.tif]

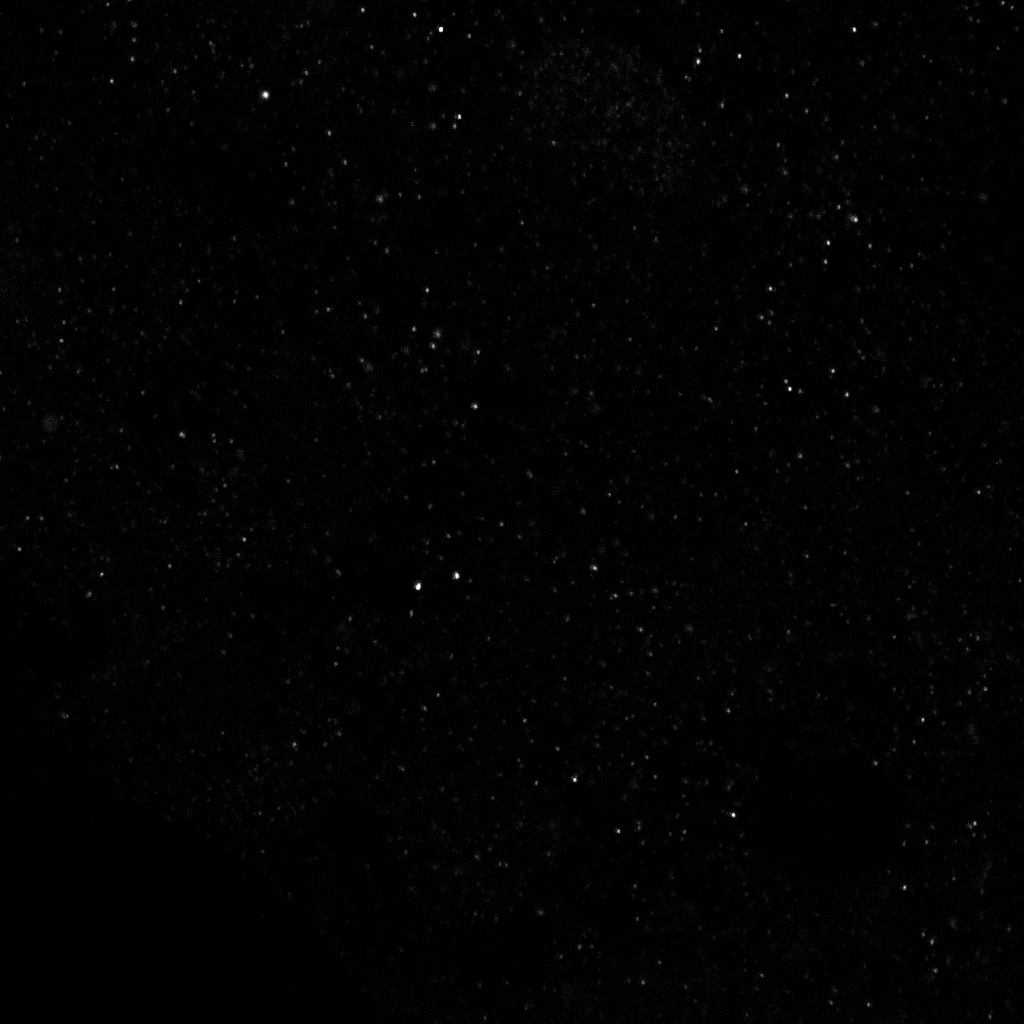

Supplement: Supplementary file 4 — Source data Fig. 2 [file 44319_2025_613_MOESM4_ESM.zip › Figure 2/H/ARPe siCLN3 1.tif]

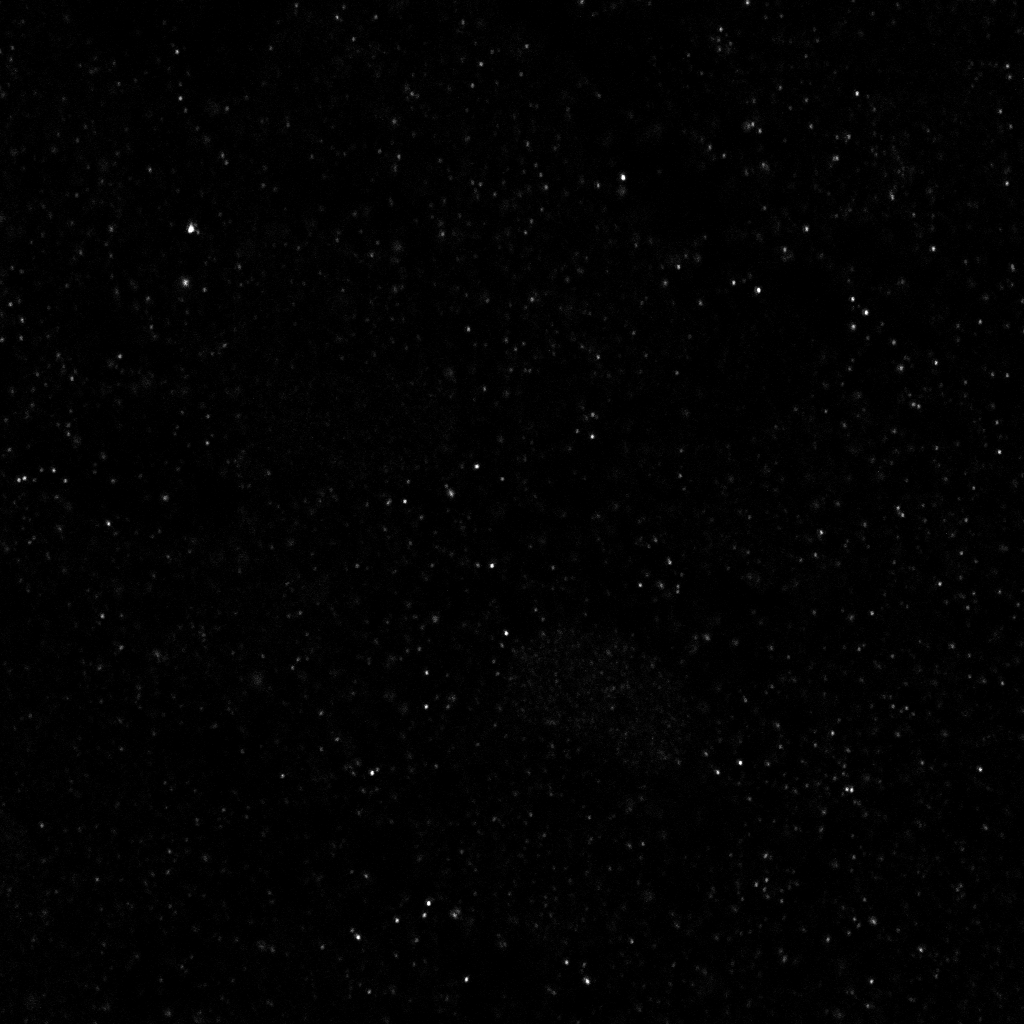

Supplement: Supplementary file 4 — Source data Fig. 2 [file 44319_2025_613_MOESM4_ESM.zip › Figure 2/H/ARPE siCLN3 2 p73.tif]

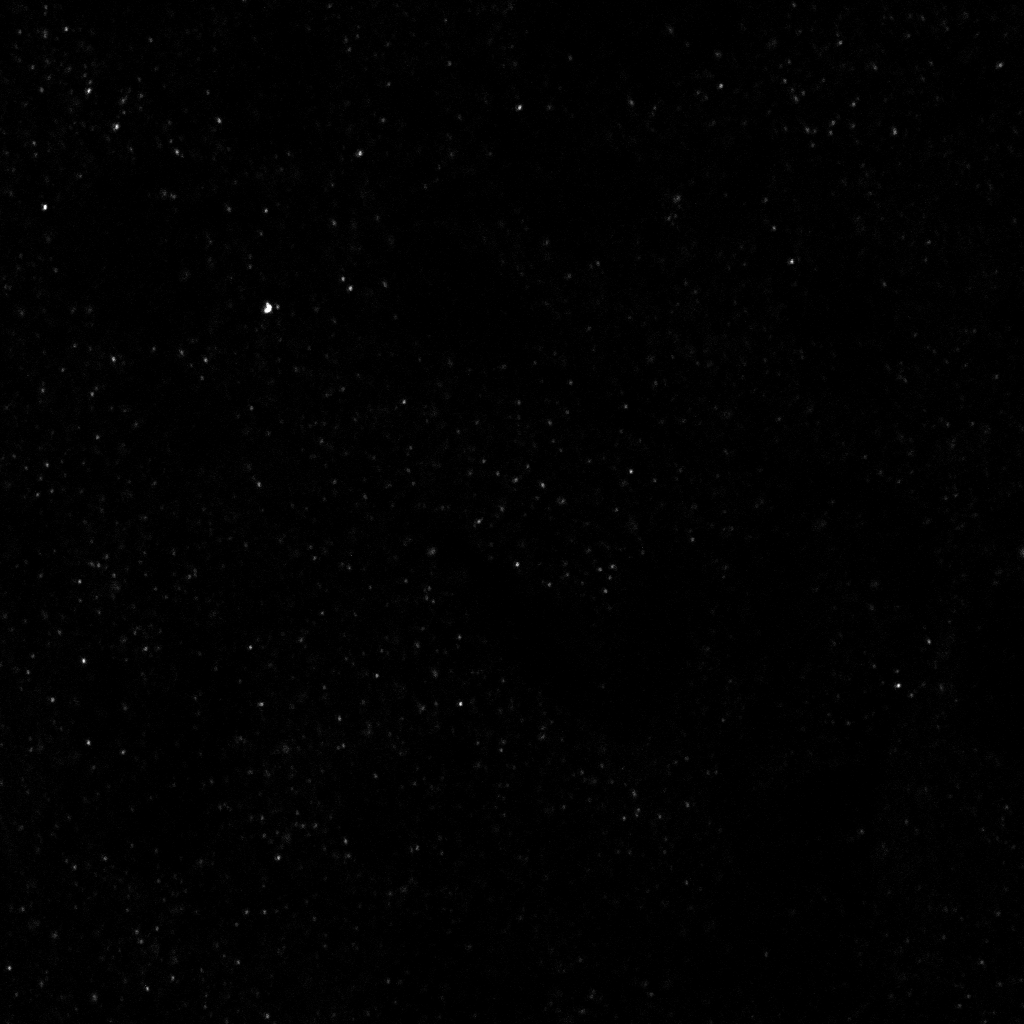

Supplement: Supplementary file 4 — Source data Fig. 2 [file 44319_2025_613_MOESM4_ESM.zip › Figure 2/H/ARPE19 siCT p73.tif]

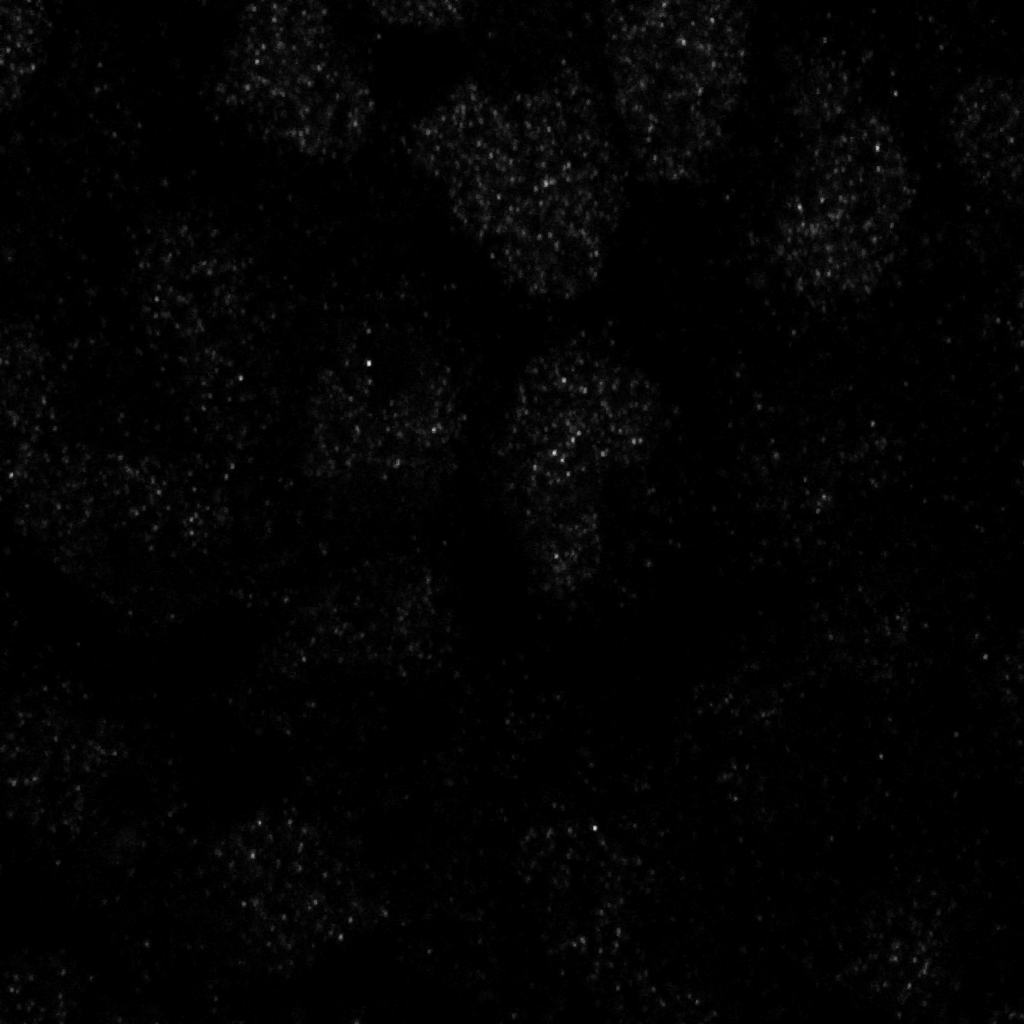

Supplement: Supplementary file 4 — Source data Fig. 2 [file 44319_2025_613_MOESM4_ESM.zip › Figure 2/H/HEK siCLN3 1 p73.tif]

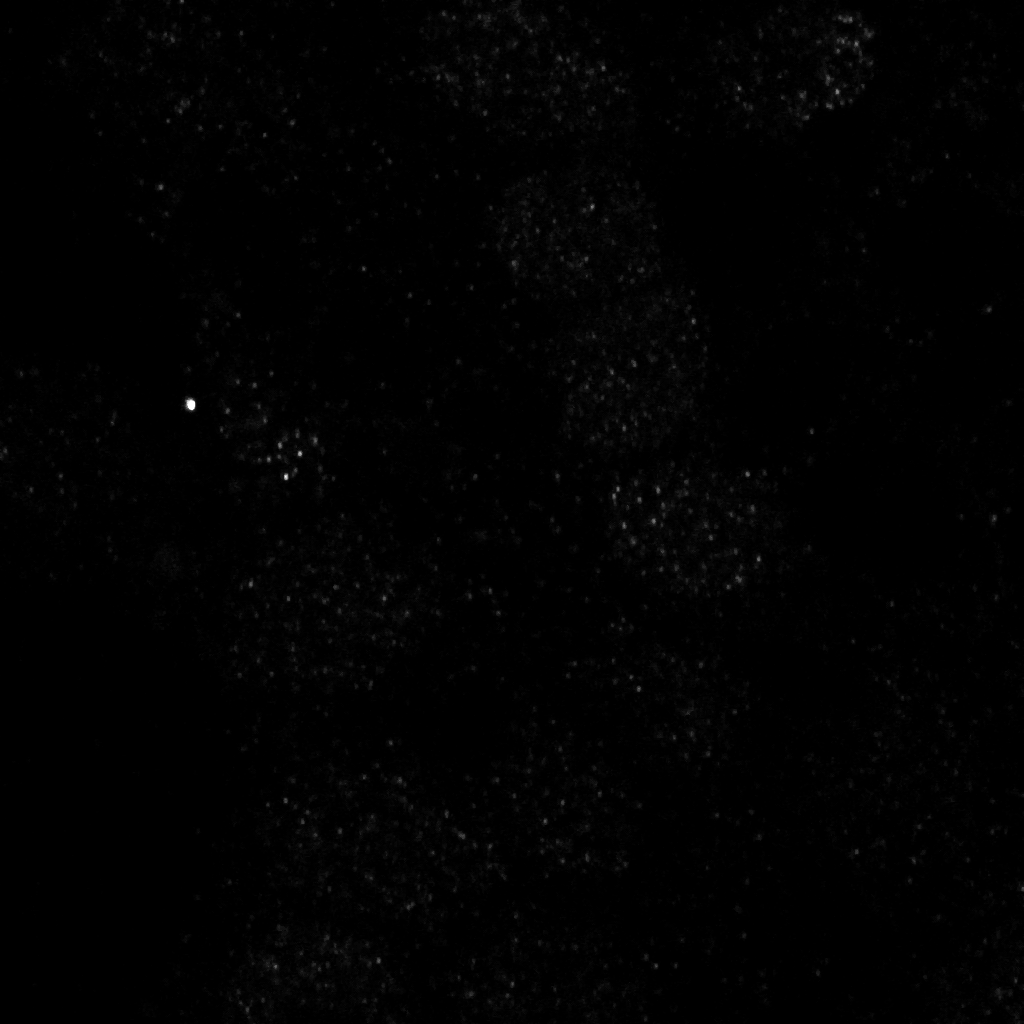

Supplement: Supplementary file 4 — Source data Fig. 2 [file 44319_2025_613_MOESM4_ESM.zip › Figure 2/H/HEK siCLN3 2 p73.tif]

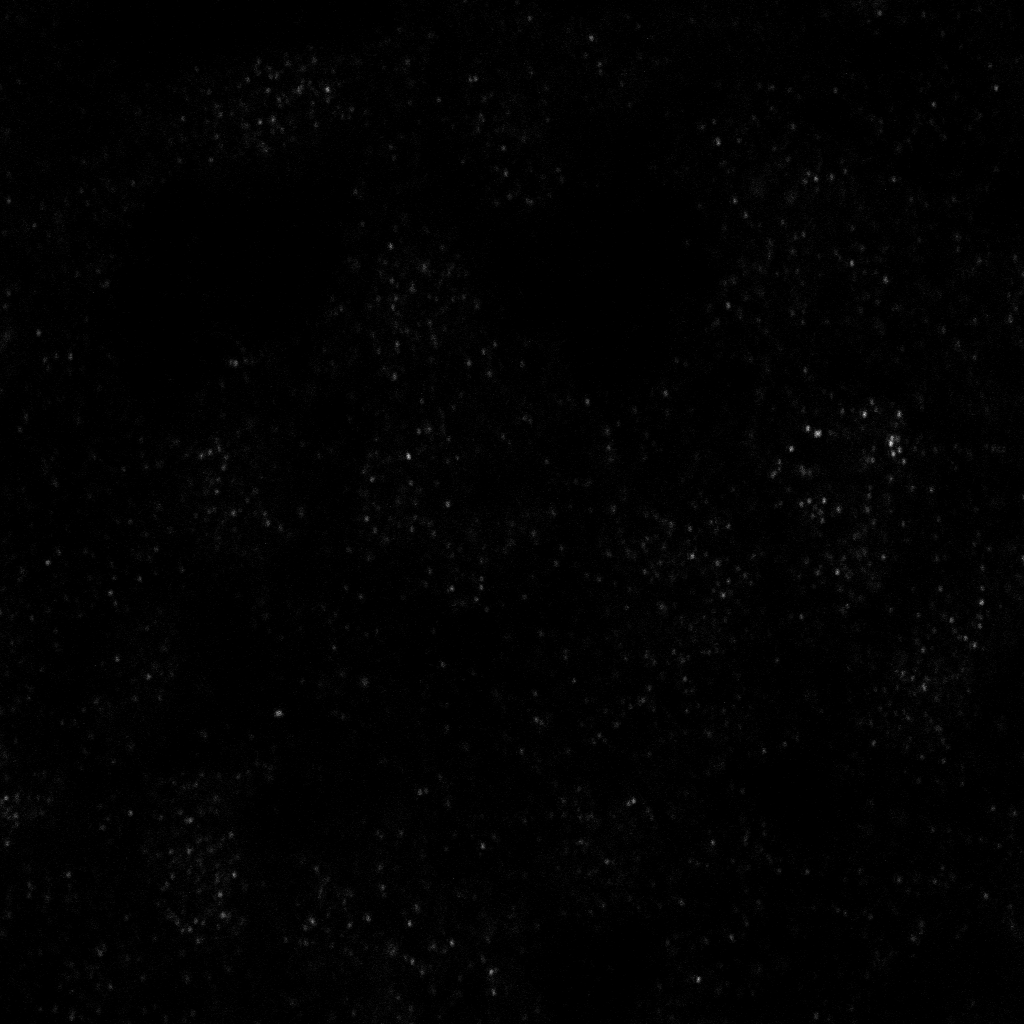

Supplement: Supplementary file 4 — Source data Fig. 2 [file 44319_2025_613_MOESM4_ESM.zip › Figure 2/H/HEK siCT p73.tif]

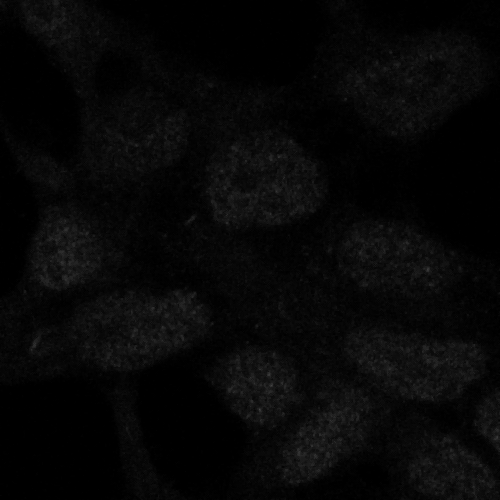

Supplement: Supplementary file 4 — Source data Fig. 2 [file 44319_2025_613_MOESM4_ESM.zip › Figure 2/I/p73_siCt.tif]

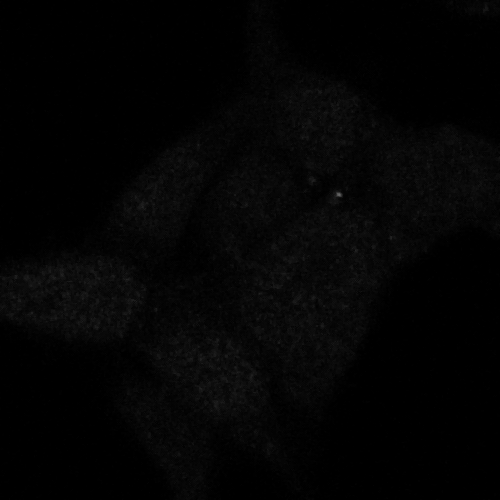

Supplement: Supplementary file 4 — Source data Fig. 2 [file 44319_2025_613_MOESM4_ESM.zip › Figure 2/I/p73_siYAP1.tif]

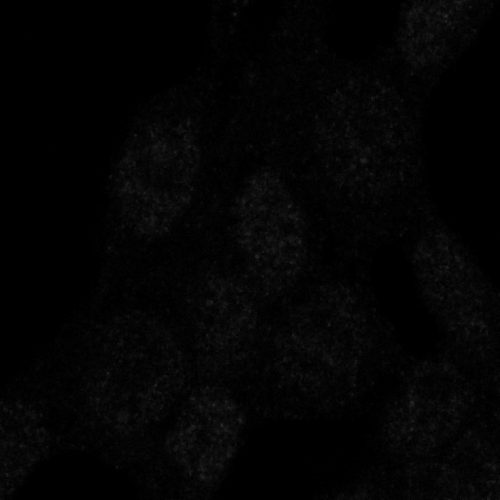

Supplement: Supplementary file 4 — Source data Fig. 2 [file 44319_2025_613_MOESM4_ESM.zip › Figure 2/I/p73_siYAP2.tif]

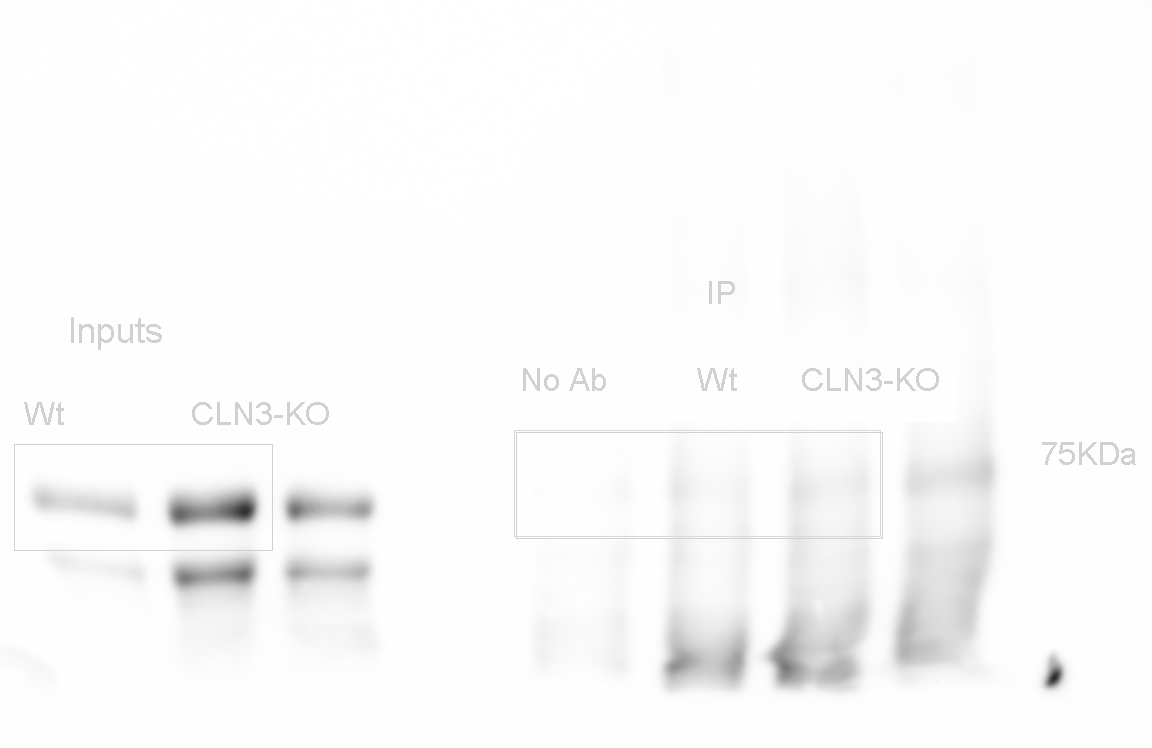

Supplement: Supplementary file 4 — Source data Fig. 2 [file 44319_2025_613_MOESM4_ESM.zip › Figure 2/J/J p73.tif]

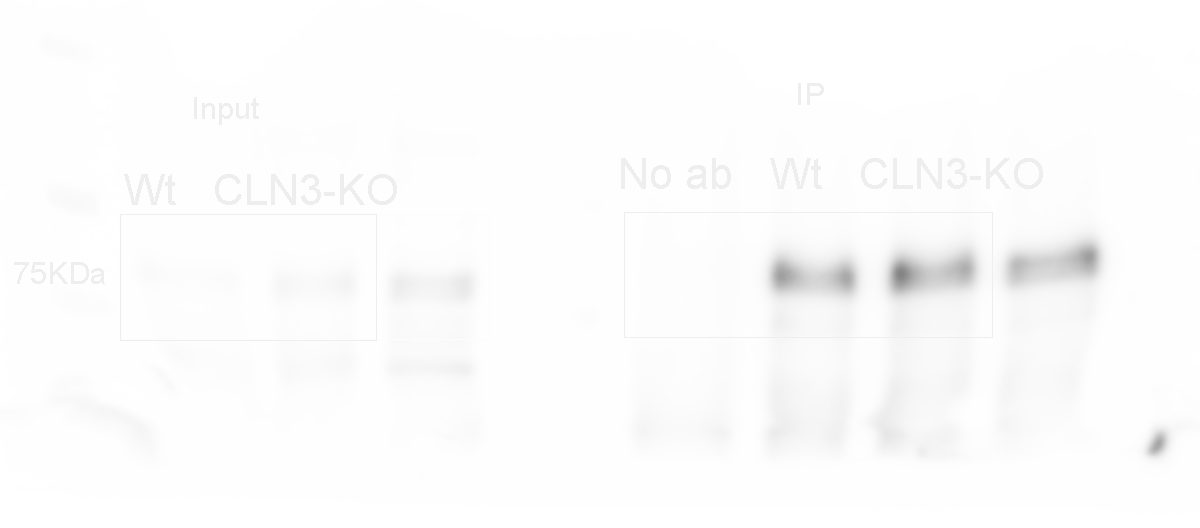

Supplement: Supplementary file 4 — Source data Fig. 2 [file 44319_2025_613_MOESM4_ESM.zip › Figure 2/J/J YAP.tif]

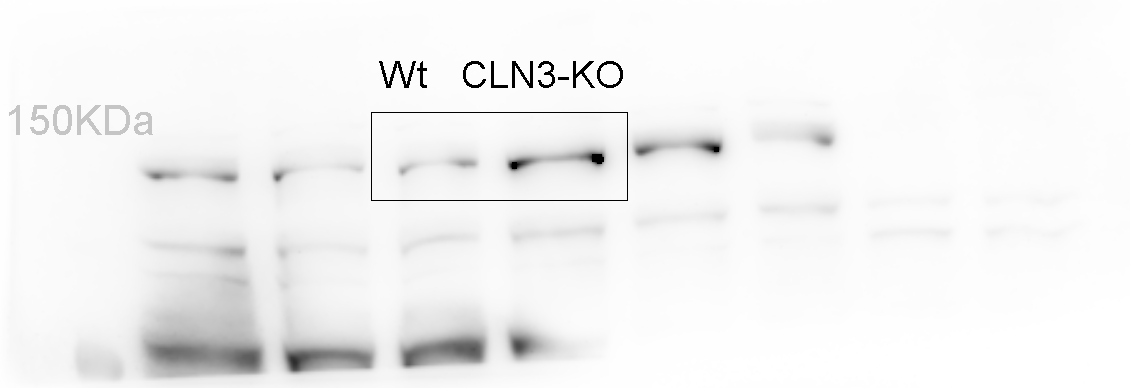

Supplement: Supplementary file 5 — Source data Fig. 3 [file 44319_2025_613_MOESM5_ESM.zip › Figure 3/A/3A cAbl.tif]

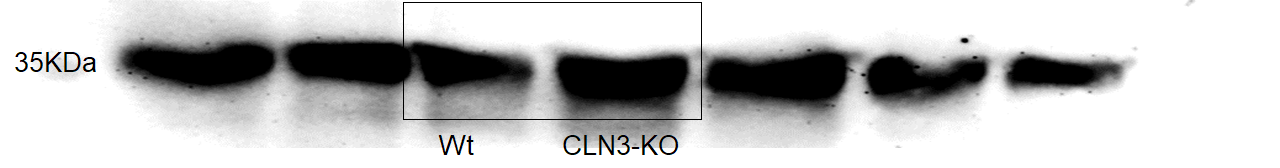

Supplement: Supplementary file 5 — Source data Fig. 3 [file 44319_2025_613_MOESM5_ESM.zip › Figure 3/A/3A GAPDH.tif]

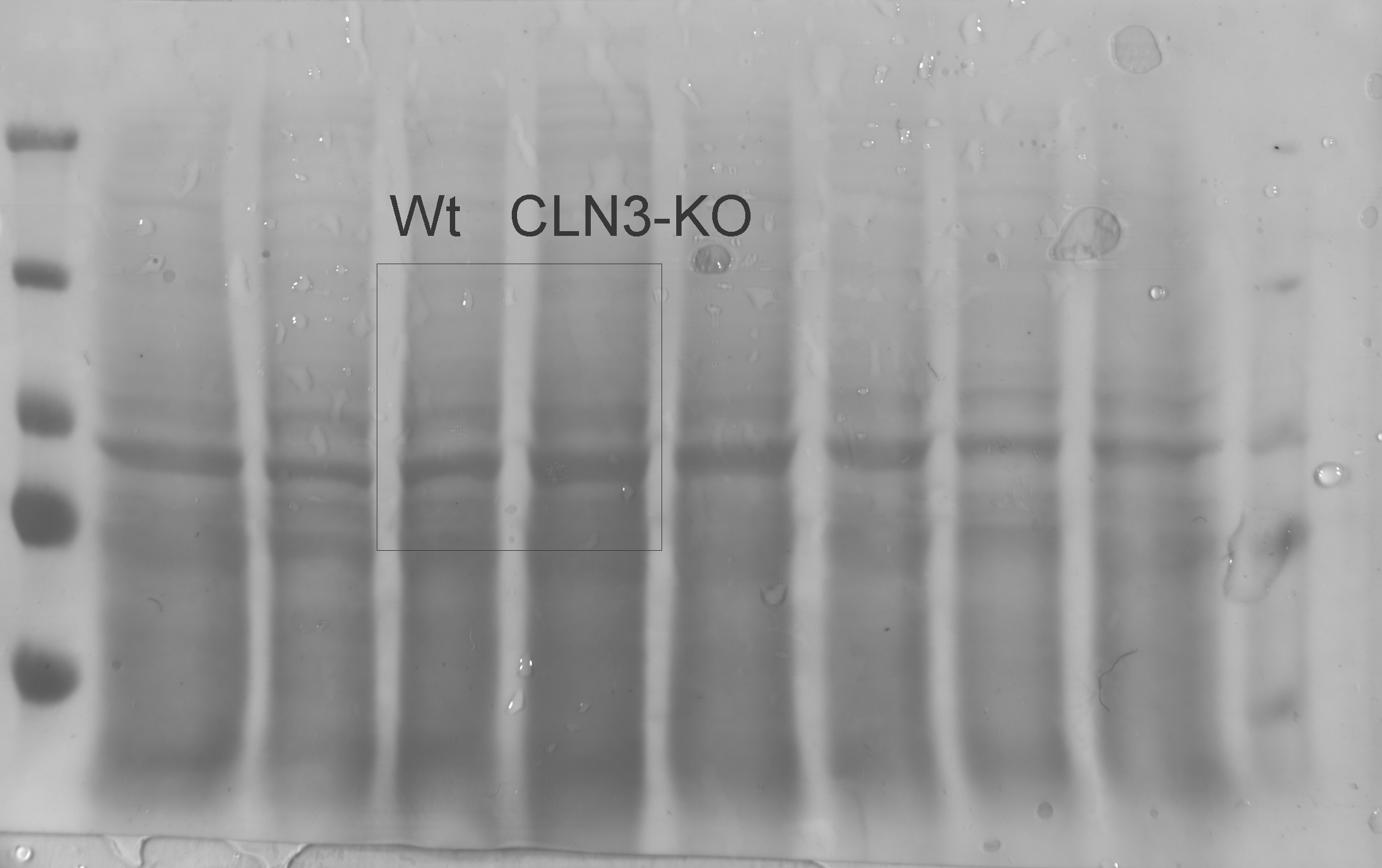

Supplement: Supplementary file 5 — Source data Fig. 3 [file 44319_2025_613_MOESM5_ESM.zip › Figure 3/A/3A ponceau.tif]

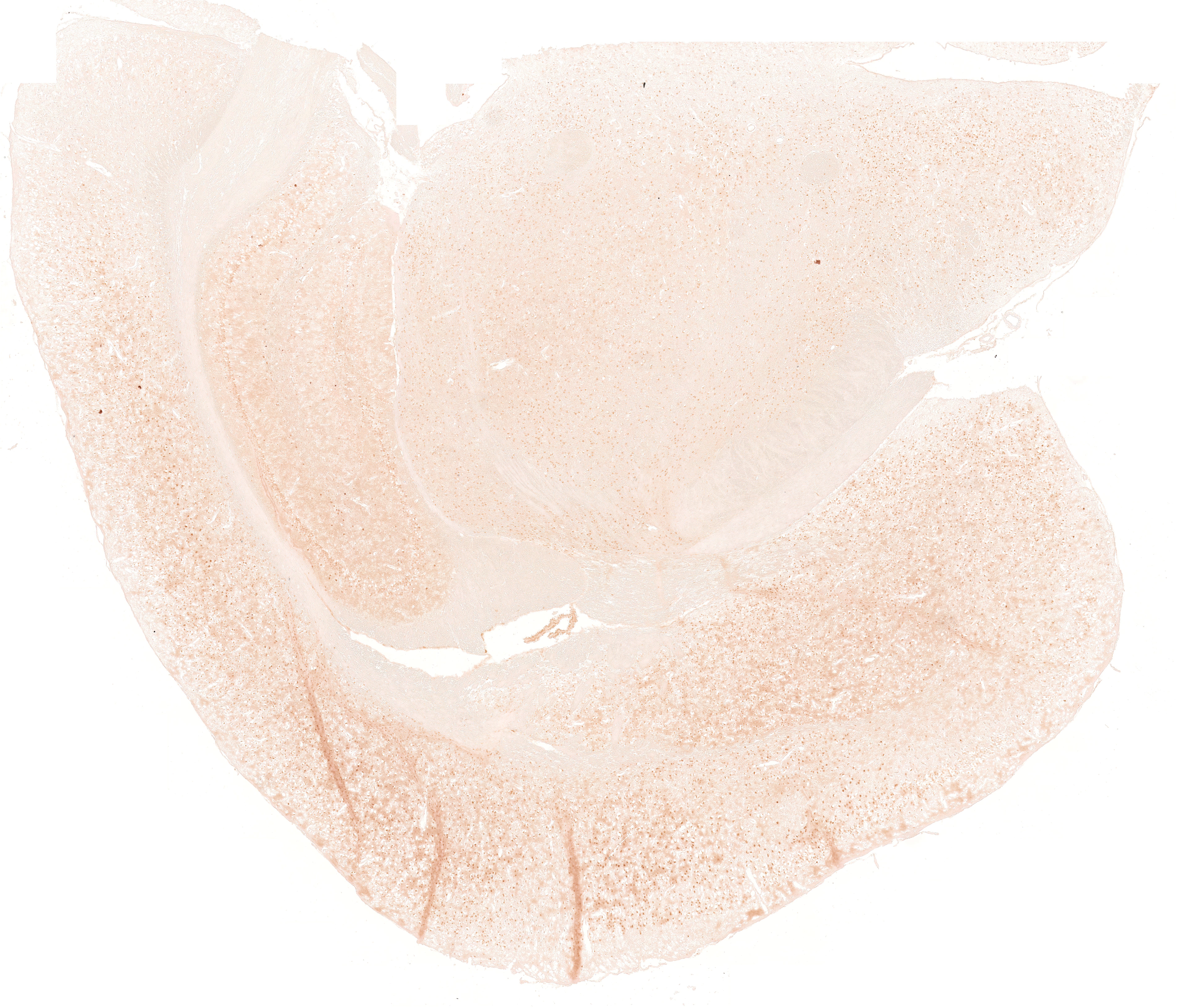

Supplement: Supplementary file 5 — Source data Fig. 3 [file 44319_2025_613_MOESM5_ESM.zip › Figure 3/C/Cln3 d7 8.png]

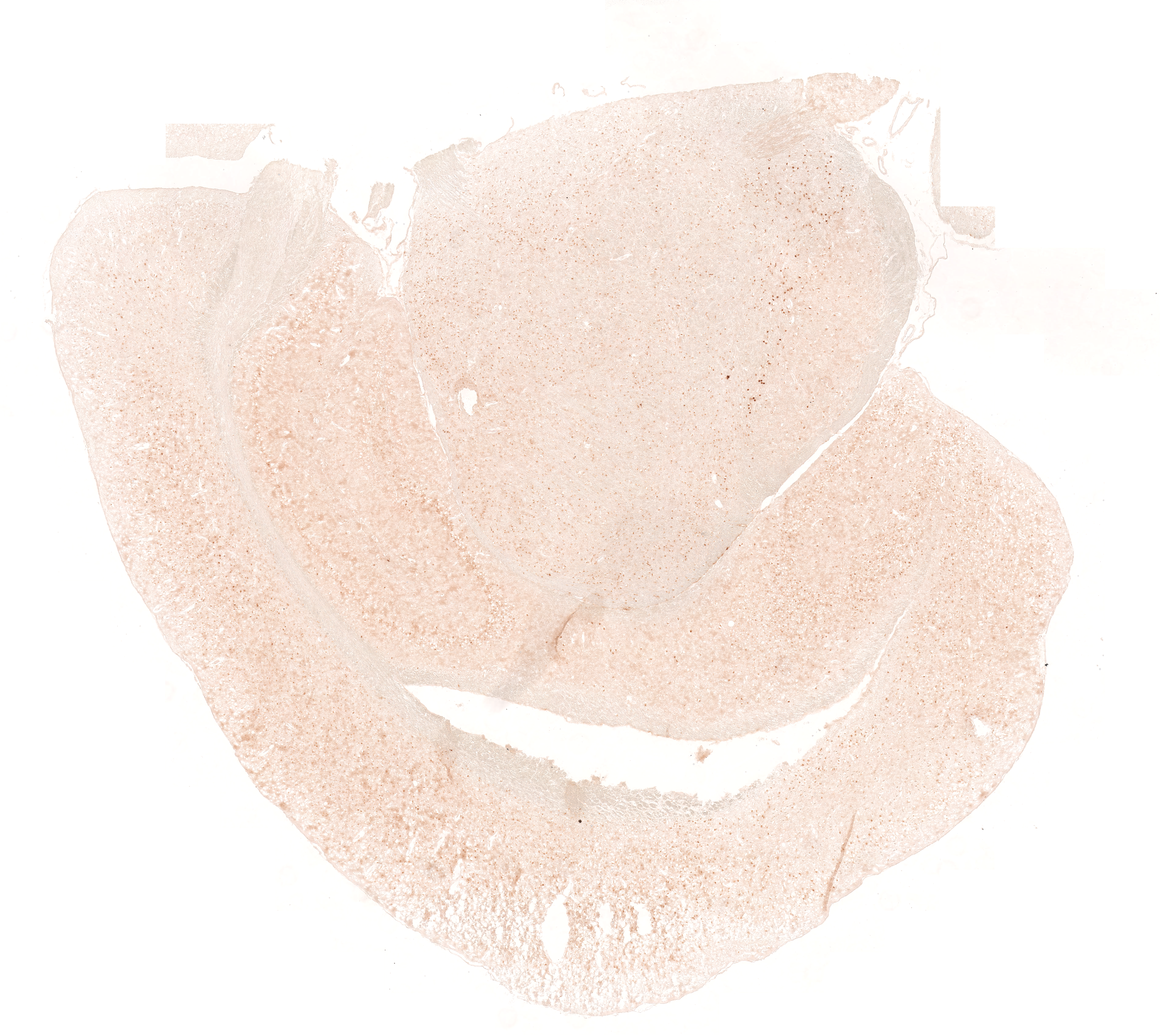

Supplement: Supplementary file 5 — Source data Fig. 3 [file 44319_2025_613_MOESM5_ESM.zip › Figure 3/C/Wt.png]

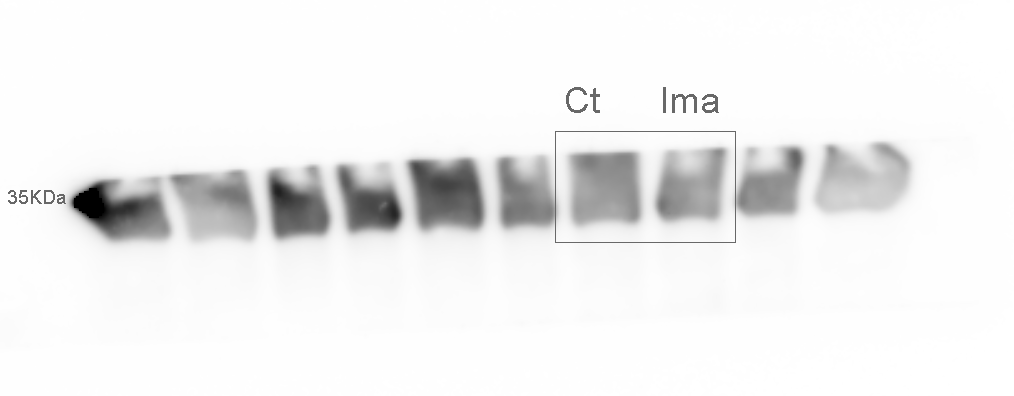

Supplement: Supplementary file 5 — Source data Fig. 3 [file 44319_2025_613_MOESM5_ESM.zip › Figure 3/D/3D GAPDH.tif]

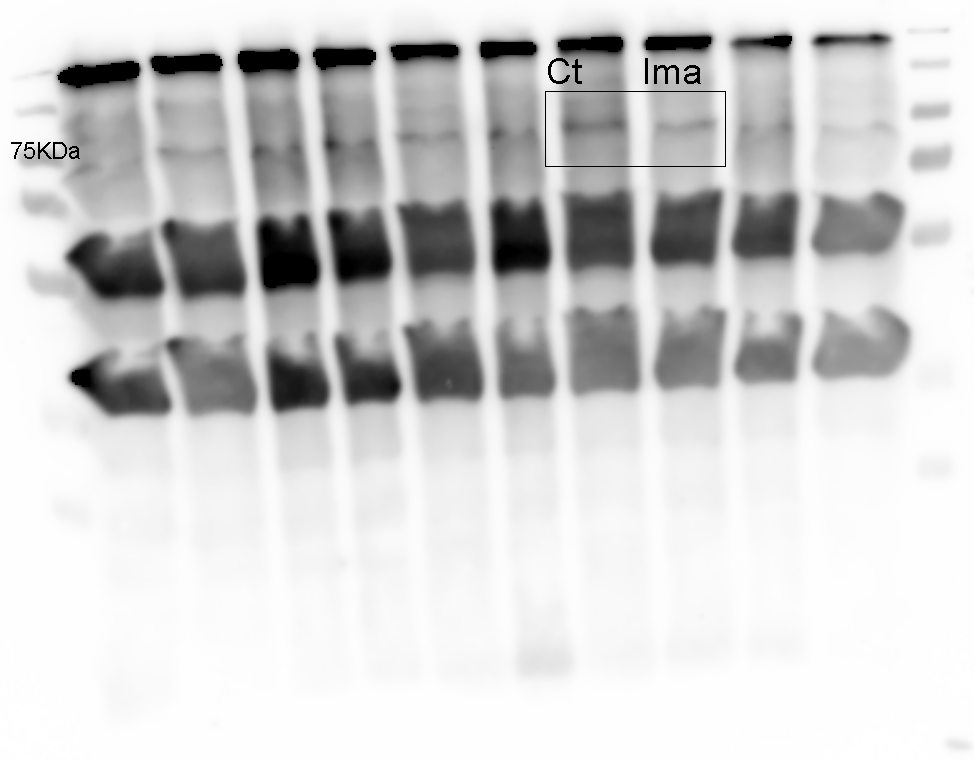

Supplement: Supplementary file 5 — Source data Fig. 3 [file 44319_2025_613_MOESM5_ESM.zip › Figure 3/D/3D pYap y357.tif]

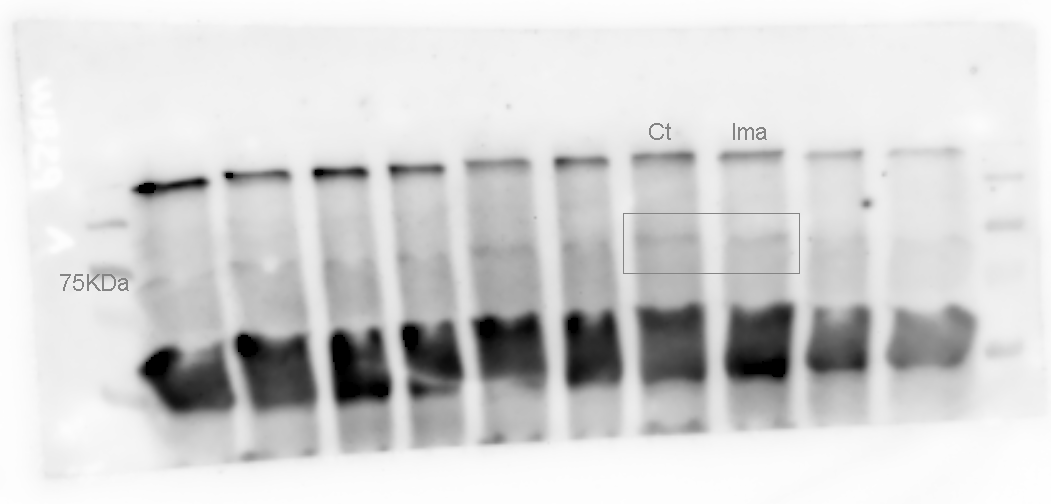

Supplement: Supplementary file 5 — Source data Fig. 3 [file 44319_2025_613_MOESM5_ESM.zip › Figure 3/D/3D Yap.tif]

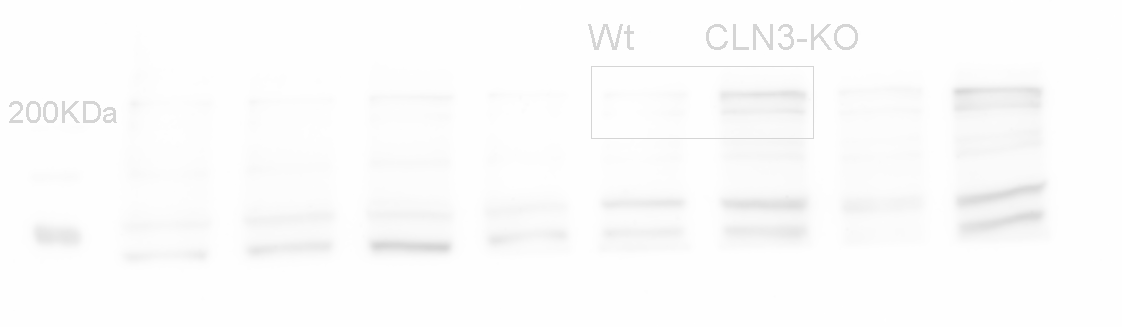

Supplement: Supplementary file 6 — Source data Fig. 4 [file 44319_2025_613_MOESM6_ESM.zip › Figure 4/B/4B ARPE ATM total.tif]

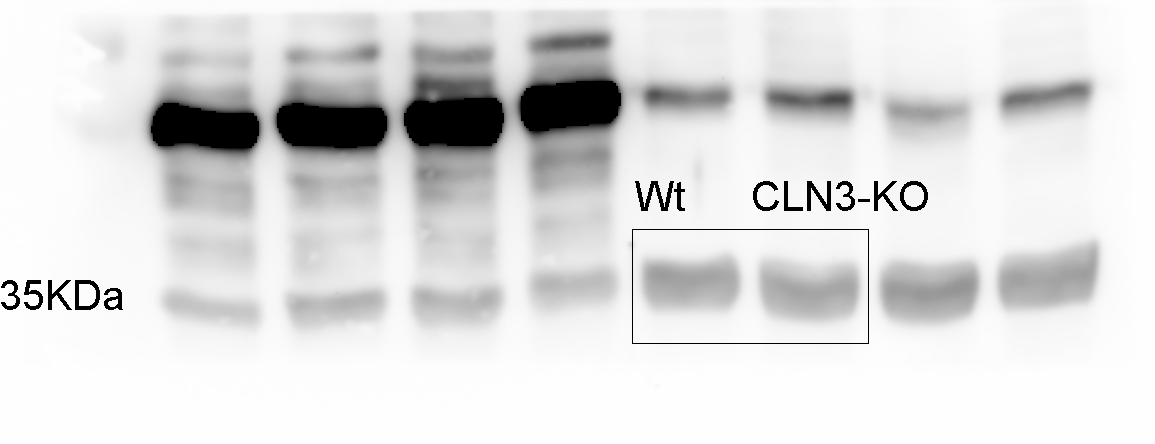

Supplement: Supplementary file 6 — Source data Fig. 4 [file 44319_2025_613_MOESM6_ESM.zip › Figure 4/B/4B ARPE GAPDH.tif]

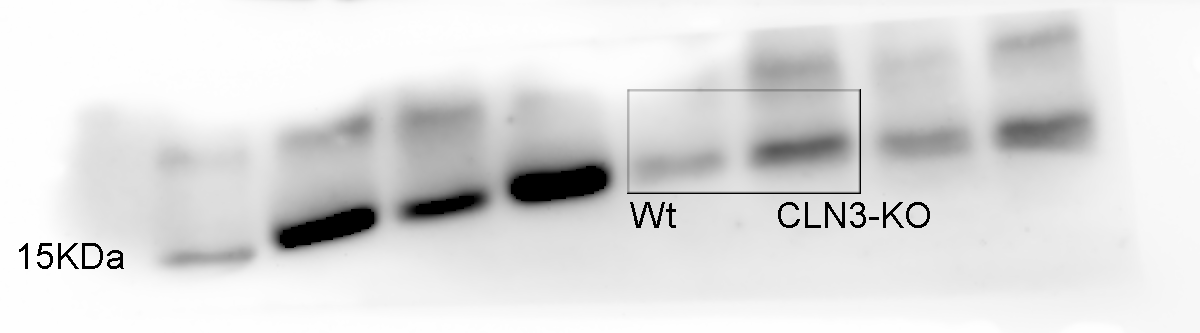

Supplement: Supplementary file 6 — Source data Fig. 4 [file 44319_2025_613_MOESM6_ESM.zip › Figure 4/B/4B ARPE H2Ax.tif]

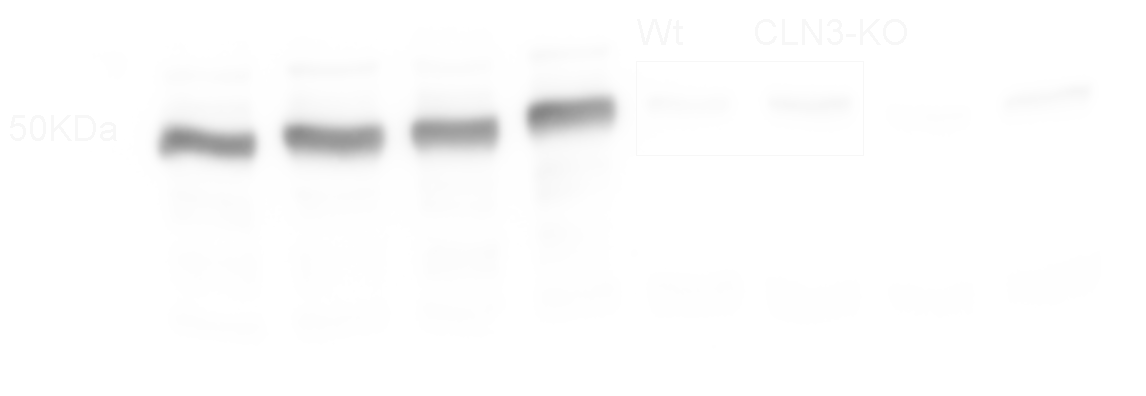

Supplement: Supplementary file 6 — Source data Fig. 4 [file 44319_2025_613_MOESM6_ESM.zip › Figure 4/B/4B ARPE p53.tif]

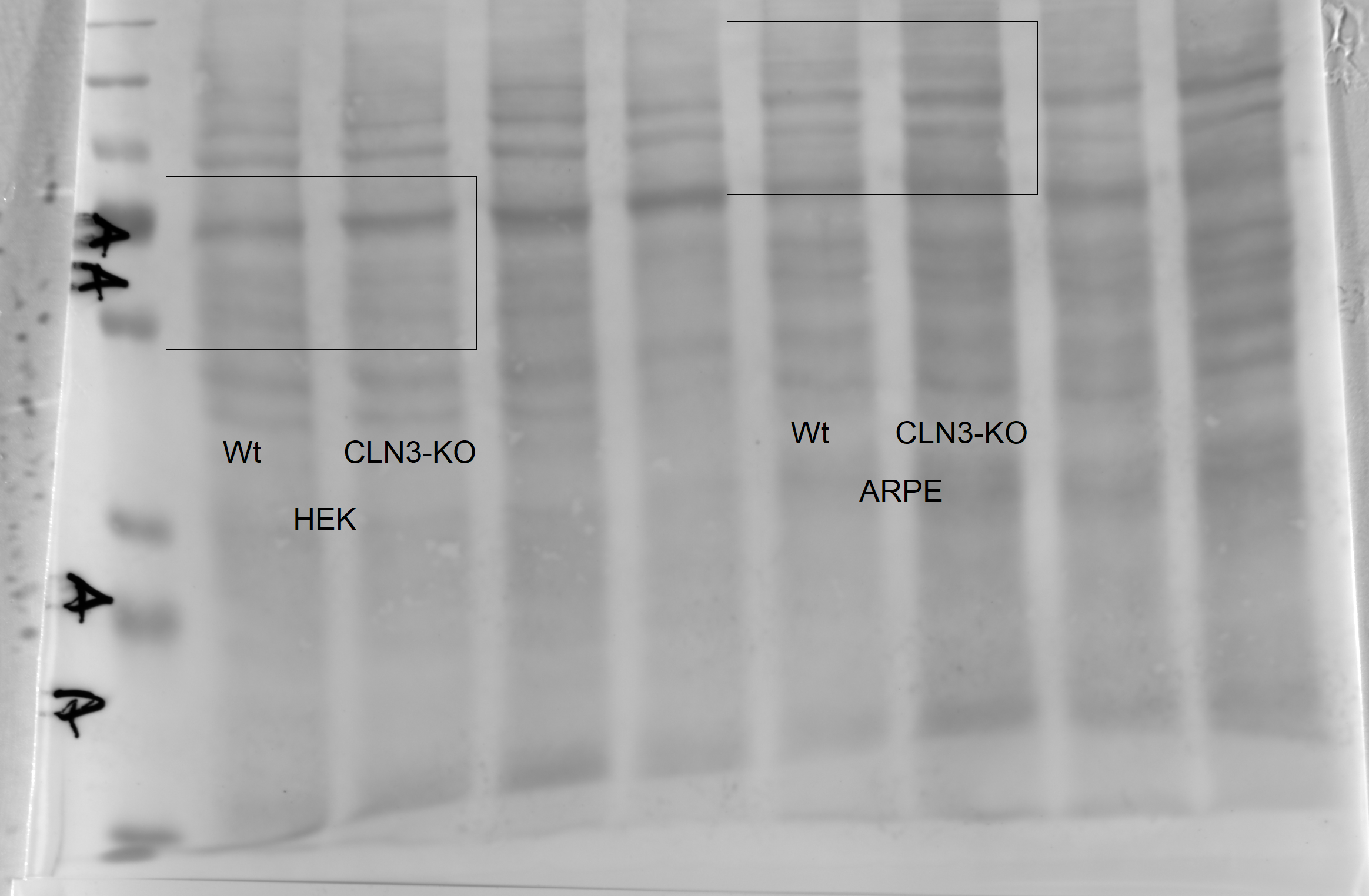

Supplement: Supplementary file 6 — Source data Fig. 4 [file 44319_2025_613_MOESM6_ESM.zip › Figure 4/B/4B HEK ARPE Ponceau.tif]

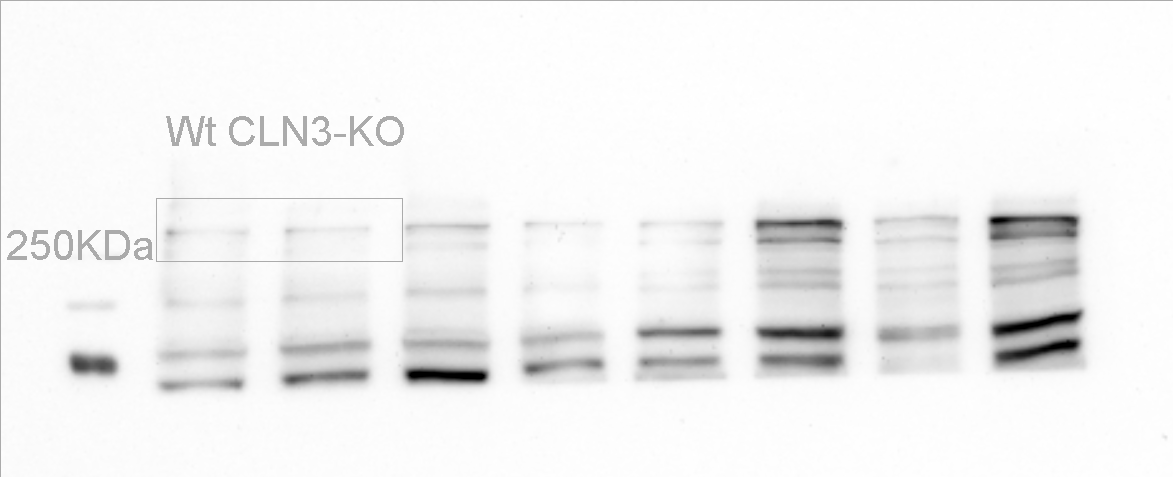

Supplement: Supplementary file 6 — Source data Fig. 4 [file 44319_2025_613_MOESM6_ESM.zip › Figure 4/B/4B HEK ATM total.tif]

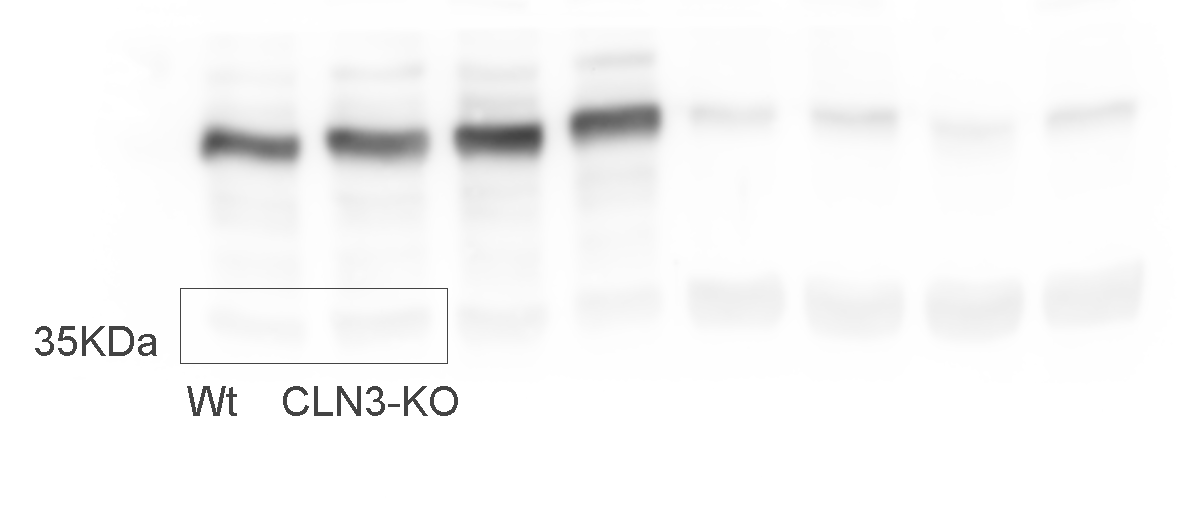

Supplement: Supplementary file 6 — Source data Fig. 4 [file 44319_2025_613_MOESM6_ESM.zip › Figure 4/B/4B HEK GAPDH.tif]

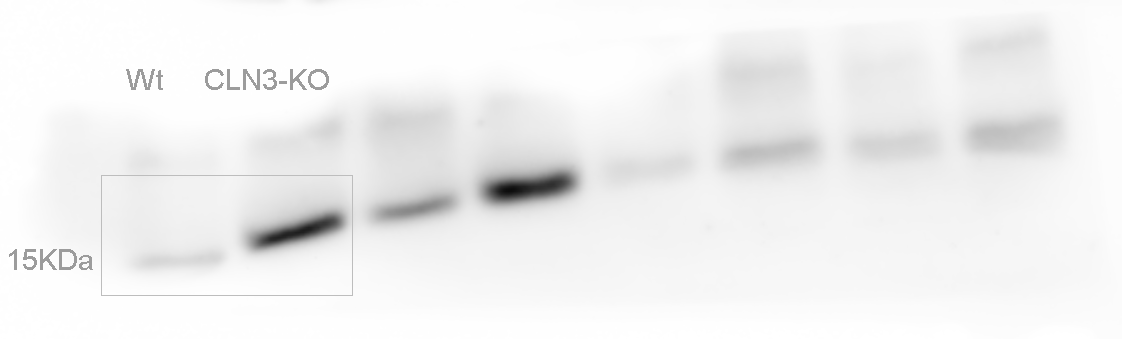

Supplement: Supplementary file 6 — Source data Fig. 4 [file 44319_2025_613_MOESM6_ESM.zip › Figure 4/B/4B HEK H2Ax.tif]

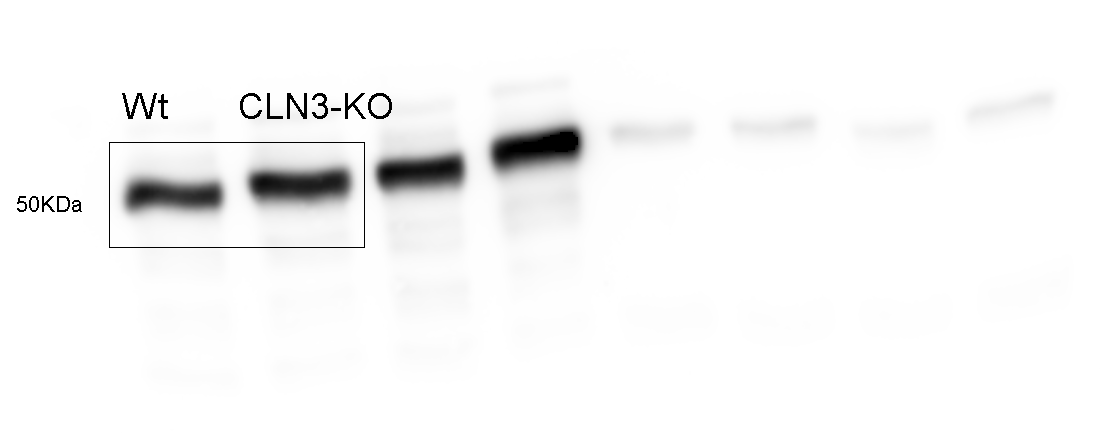

Supplement: Supplementary file 6 — Source data Fig. 4 [file 44319_2025_613_MOESM6_ESM.zip › Figure 4/B/4B HEK p53.tif]

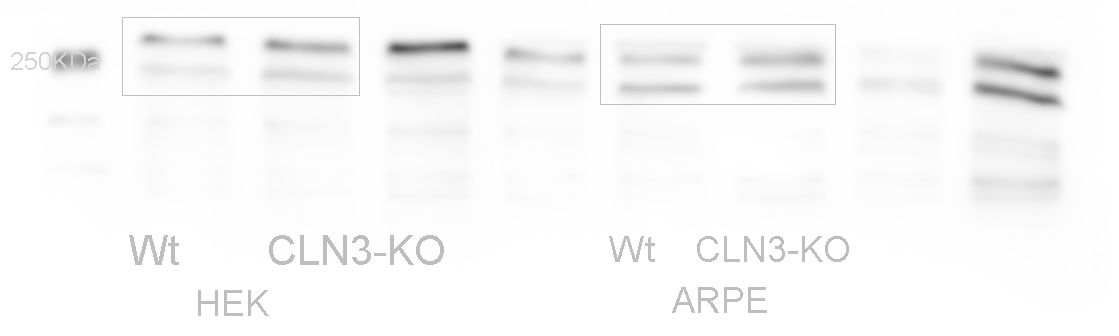

Supplement: Supplementary file 6 — Source data Fig. 4 [file 44319_2025_613_MOESM6_ESM.zip › Figure 4/B/4B pATM HEK ARPE.tif]

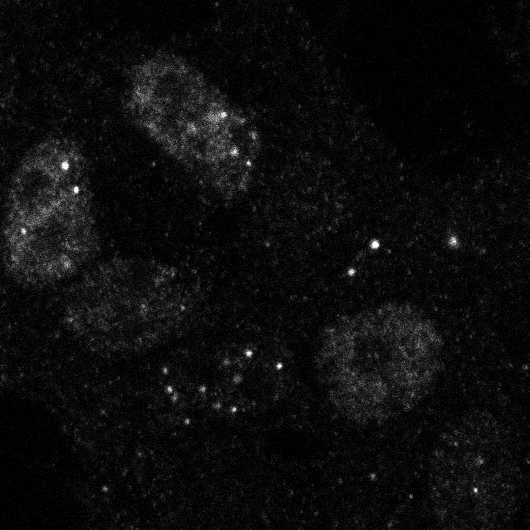

Supplement: Supplementary file 6 — Source data Fig. 4 [file 44319_2025_613_MOESM6_ESM.zip › Figure 4/C/HEK CLN3-KO p53.tif]

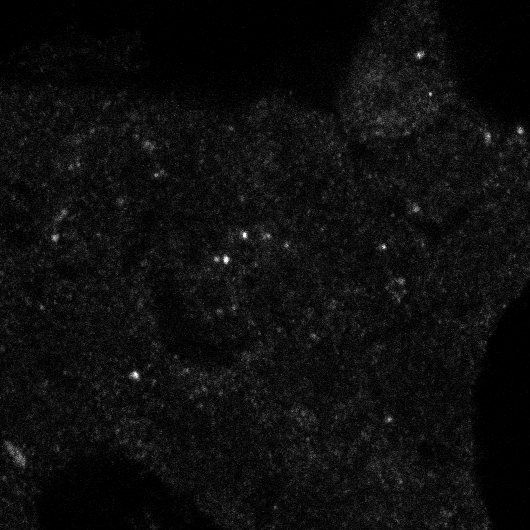

Supplement: Supplementary file 6 — Source data Fig. 4 [file 44319_2025_613_MOESM6_ESM.zip › Figure 4/C/HEK Wt p53.tif]

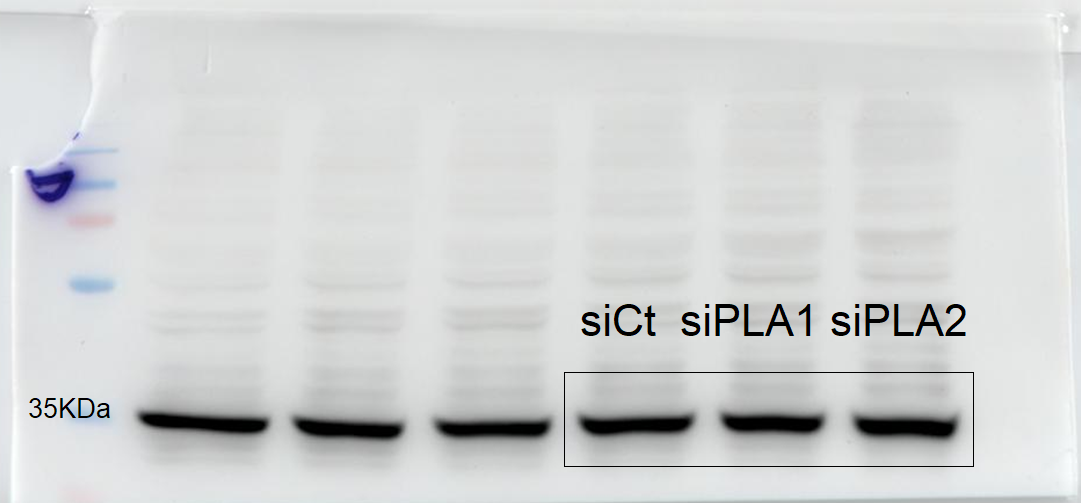

Supplement: Supplementary file 6 — Source data Fig. 4 [file 44319_2025_613_MOESM6_ESM.zip › Figure 4/E/4E GAPDH.tif]

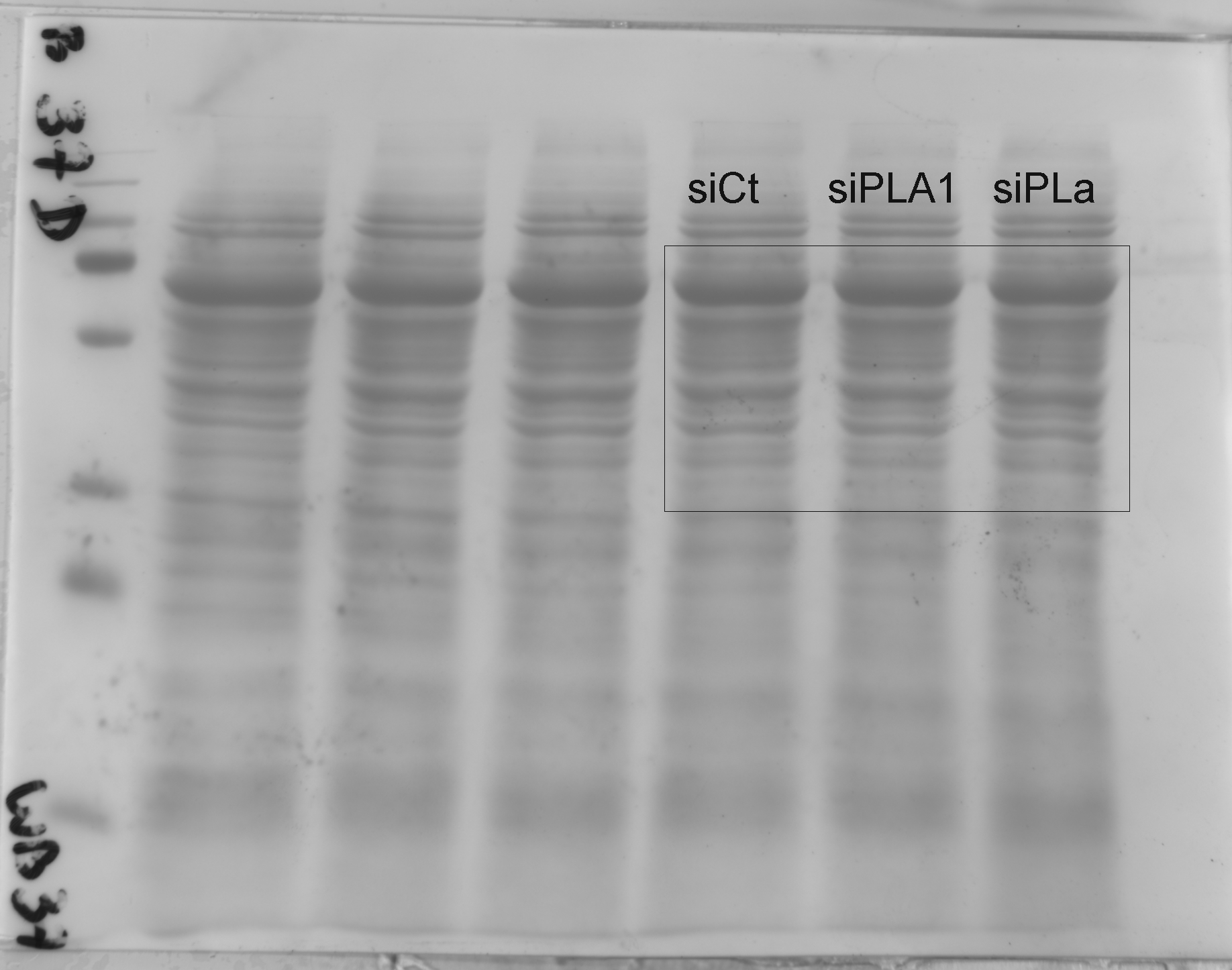

Supplement: Supplementary file 6 — Source data Fig. 4 [file 44319_2025_613_MOESM6_ESM.zip › Figure 4/E/4E ponceau.tif]

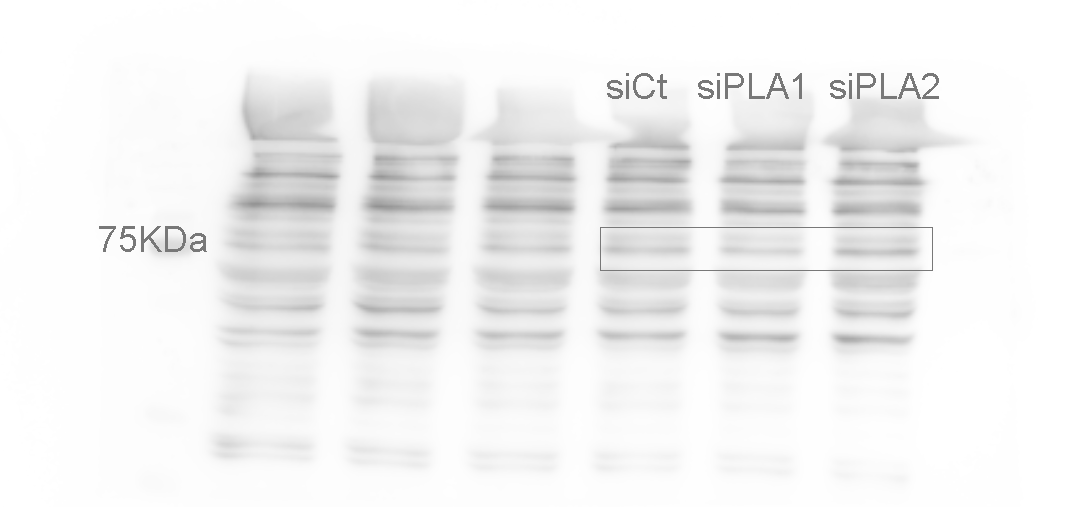

Supplement: Supplementary file 6 — Source data Fig. 4 [file 44319_2025_613_MOESM6_ESM.zip › Figure 4/E/4E pYap y357.tif]

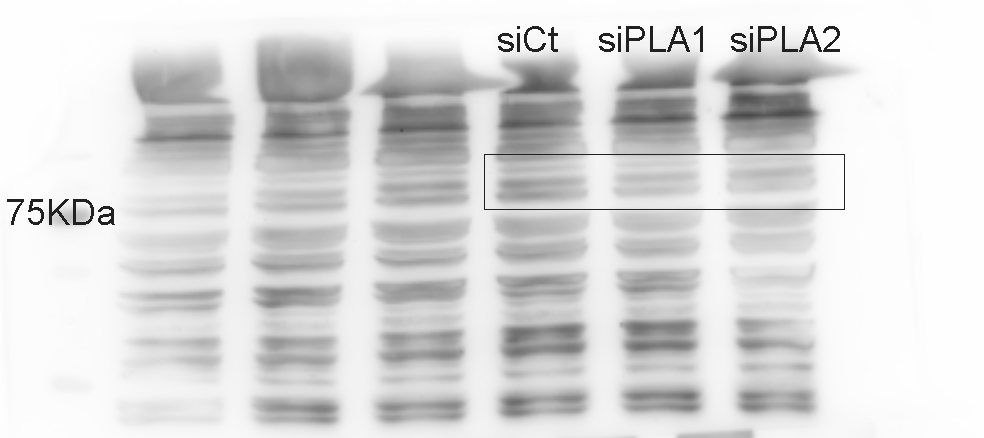

Supplement: Supplementary file 6 — Source data Fig. 4 [file 44319_2025_613_MOESM6_ESM.zip › Figure 4/E/4E Yap.tif]

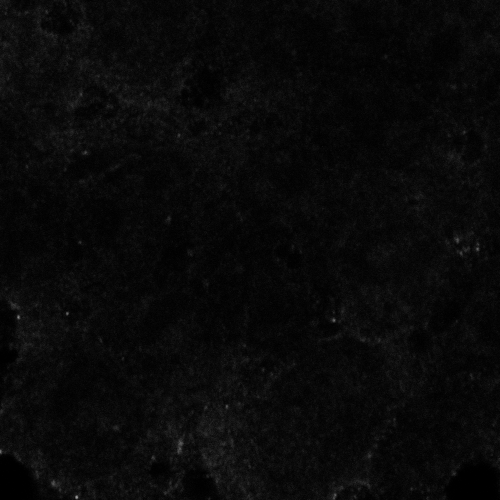

Supplement: Supplementary file 6 — Source data Fig. 4 [file 44319_2025_613_MOESM6_ESM.zip › Figure 4/F/HEK siCt pYap Y357.tif]

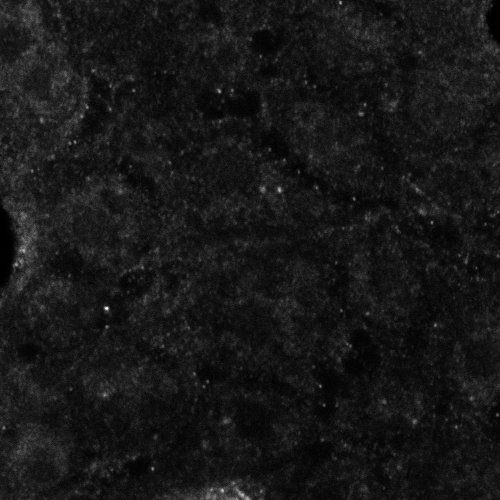

Supplement: Supplementary file 6 — Source data Fig. 4 [file 44319_2025_613_MOESM6_ESM.zip › Figure 4/F/HEK siPLA 1 pYap Y357.tif]

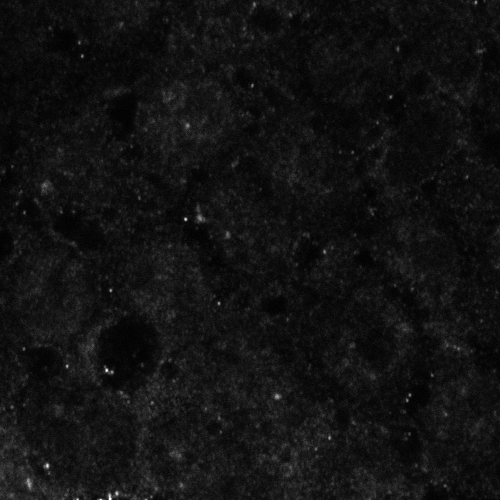

Supplement: Supplementary file 6 — Source data Fig. 4 [file 44319_2025_613_MOESM6_ESM.zip › Figure 4/F/HEK siPLA 2 pYap Y357 .tif]

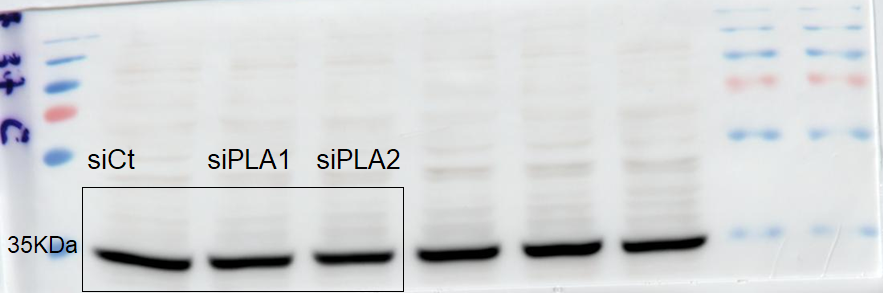

Supplement: Supplementary file 6 — Source data Fig. 4 [file 44319_2025_613_MOESM6_ESM.zip › Figure 4/G/4G GAPDH.tif]

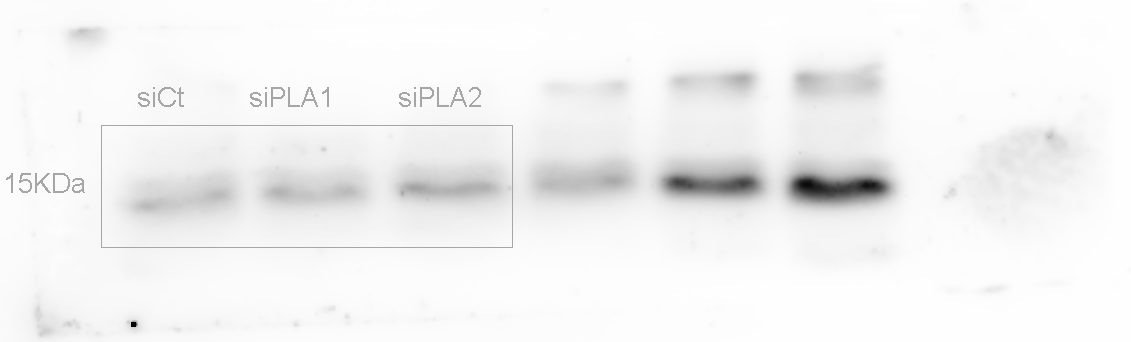

Supplement: Supplementary file 6 — Source data Fig. 4 [file 44319_2025_613_MOESM6_ESM.zip › Figure 4/G/4G H2Ax.tif]

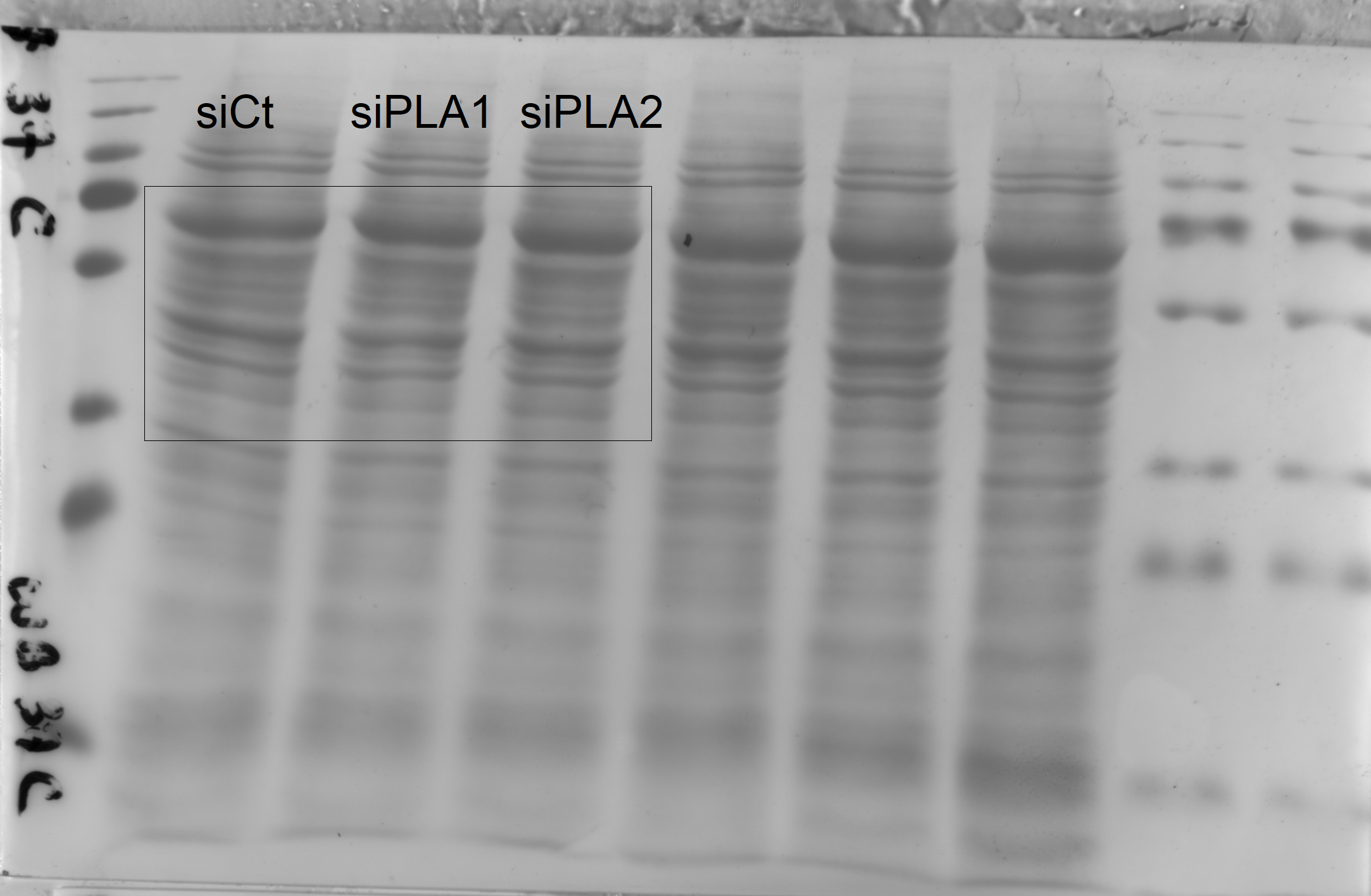

Supplement: Supplementary file 6 — Source data Fig. 4 [file 44319_2025_613_MOESM6_ESM.zip › Figure 4/G/4G ponceau.tif]

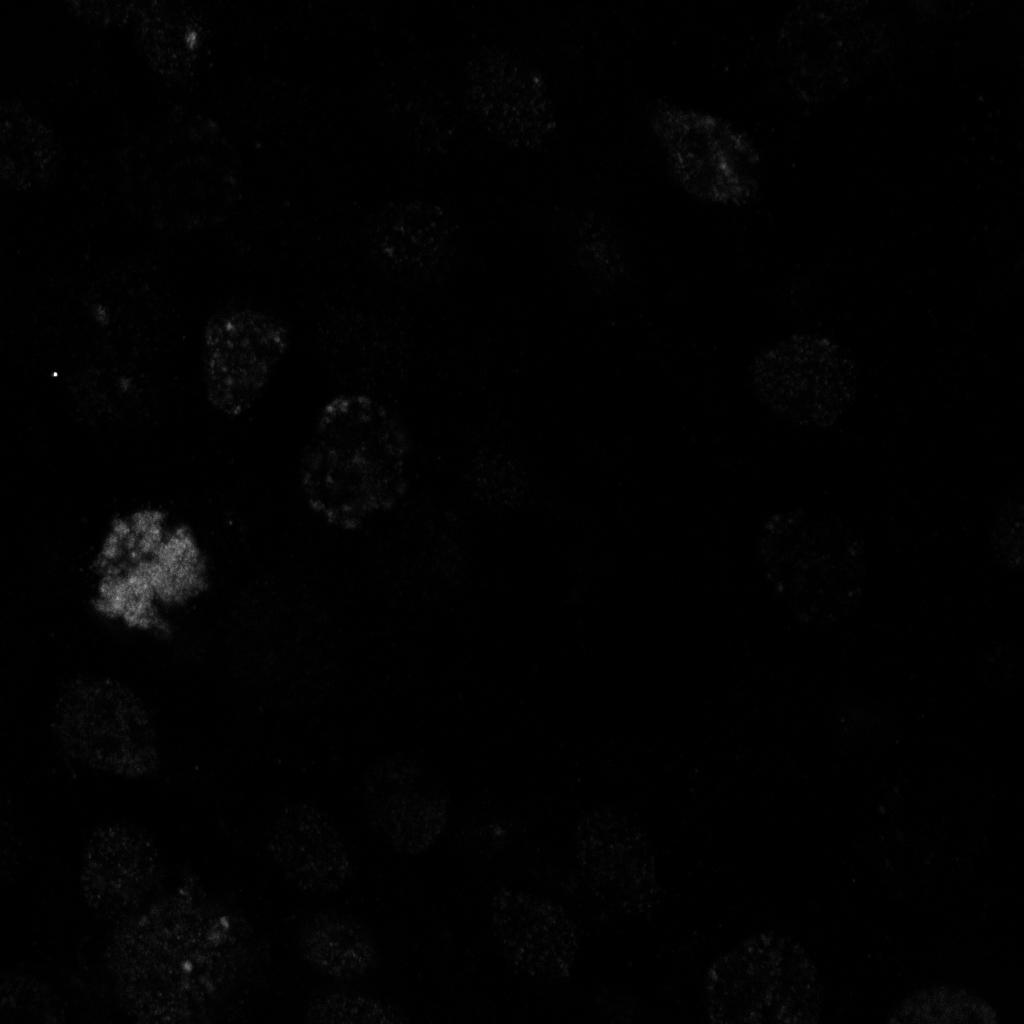

Supplement: Supplementary file 6 — Source data Fig. 4 [file 44319_2025_613_MOESM6_ESM.zip › Figure 4/H/HEK siCt p53.tif]

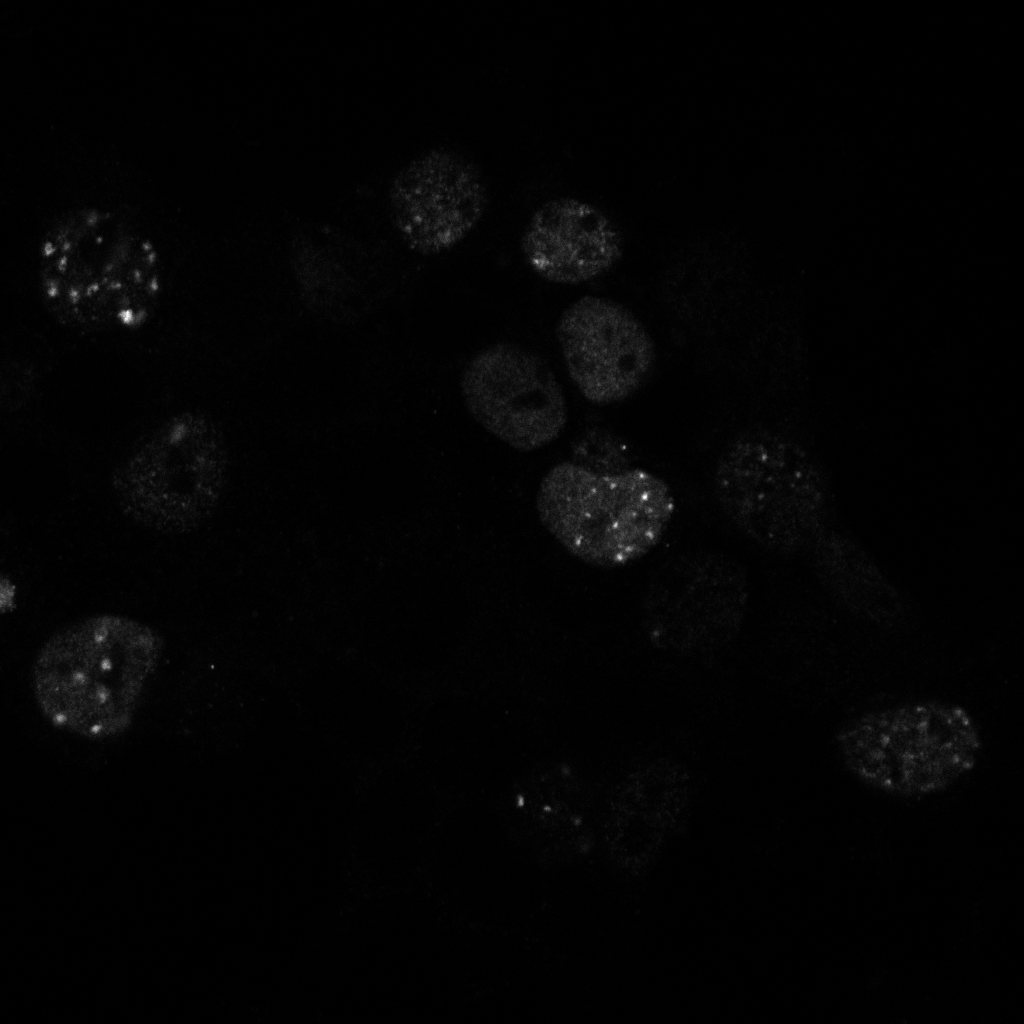

Supplement: Supplementary file 6 — Source data Fig. 4 [file 44319_2025_613_MOESM6_ESM.zip › Figure 4/H/HEK siPLA p53.tif]

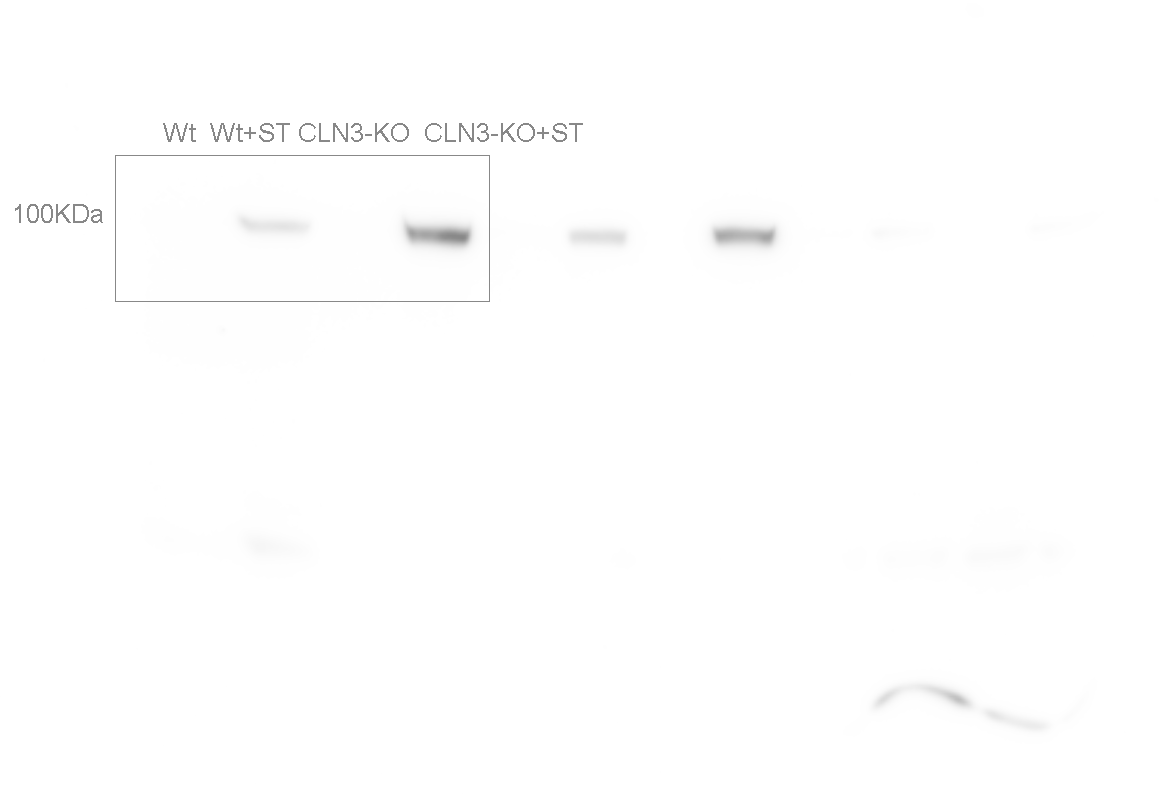

Supplement: Supplementary file 6 — Source data Fig. 4 [file 44319_2025_613_MOESM6_ESM.zip › Figure 4/J/4J cleav-PARP.tif]

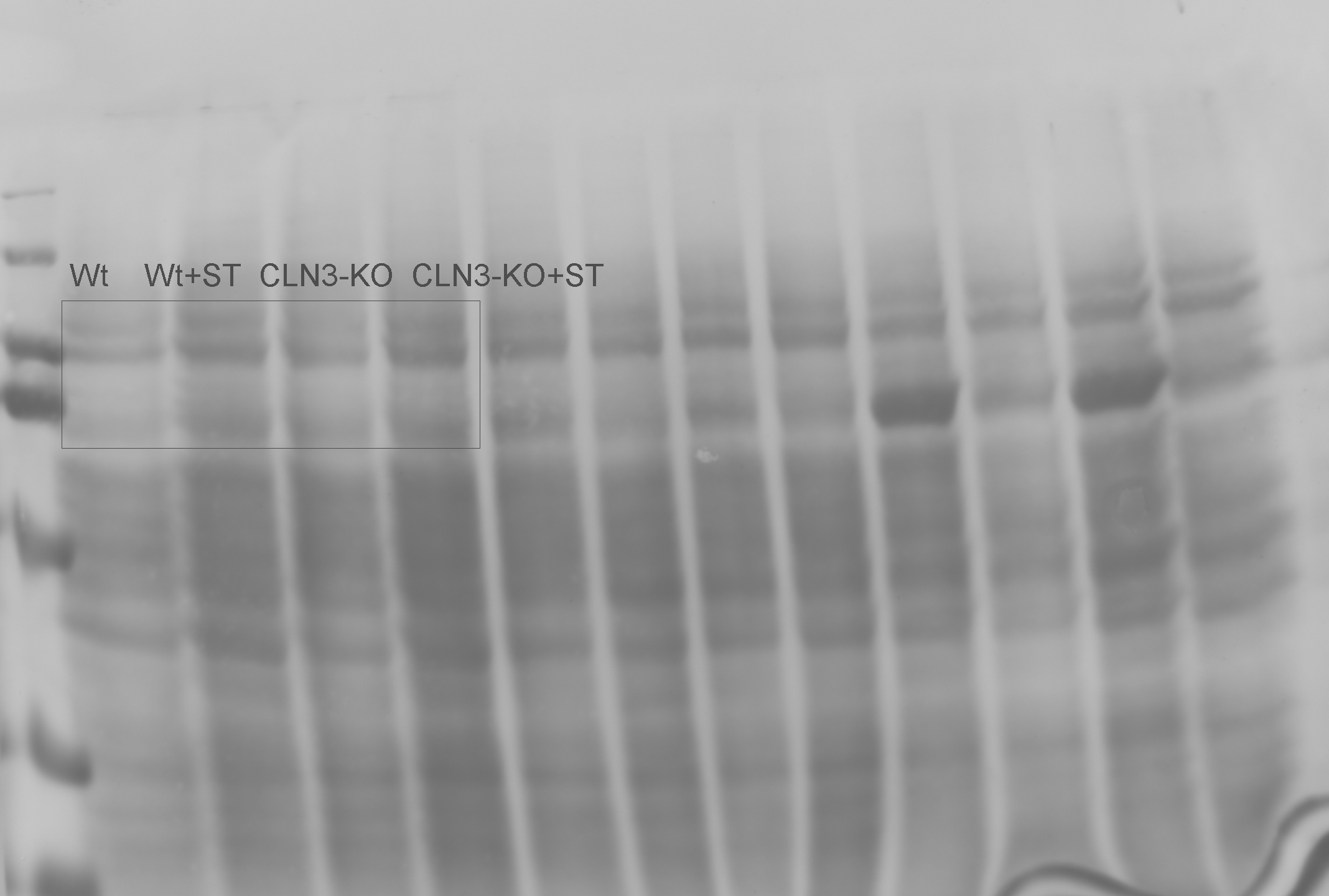

Supplement: Supplementary file 6 — Source data Fig. 4 [file 44319_2025_613_MOESM6_ESM.zip › Figure 4/J/4J ponceau.tif]

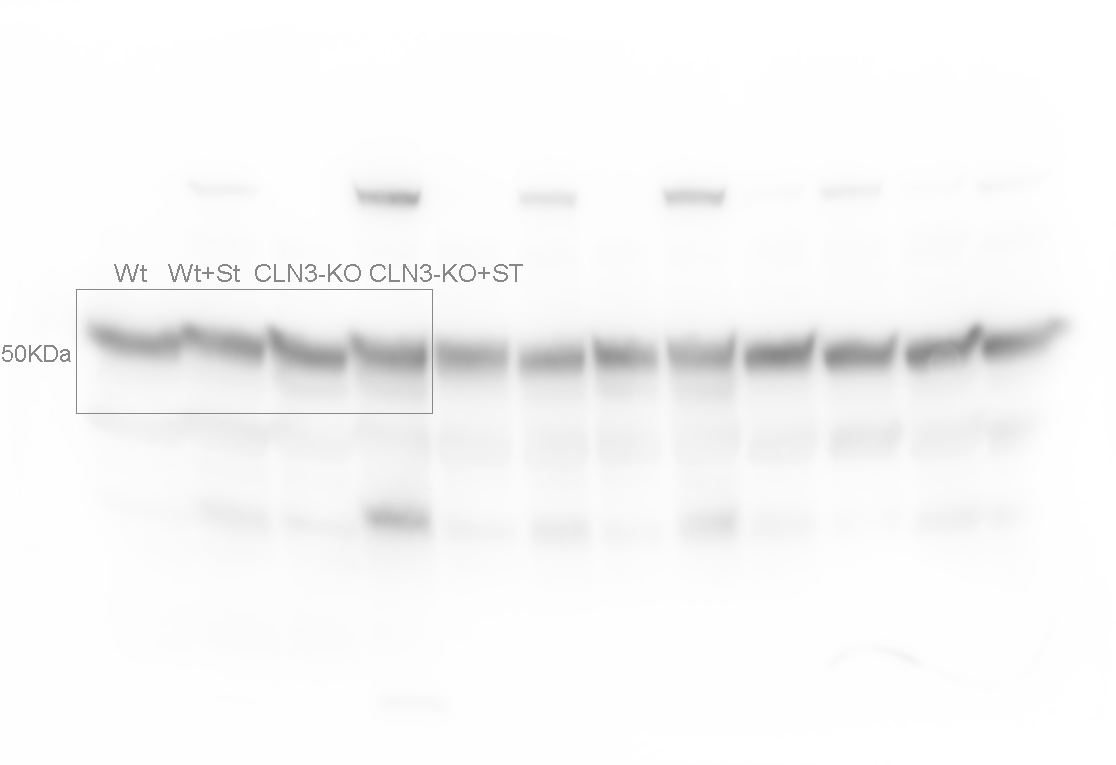

Supplement: Supplementary file 6 — Source data Fig. 4 [file 44319_2025_613_MOESM6_ESM.zip › Figure 4/J/4J Tubulin.tif]

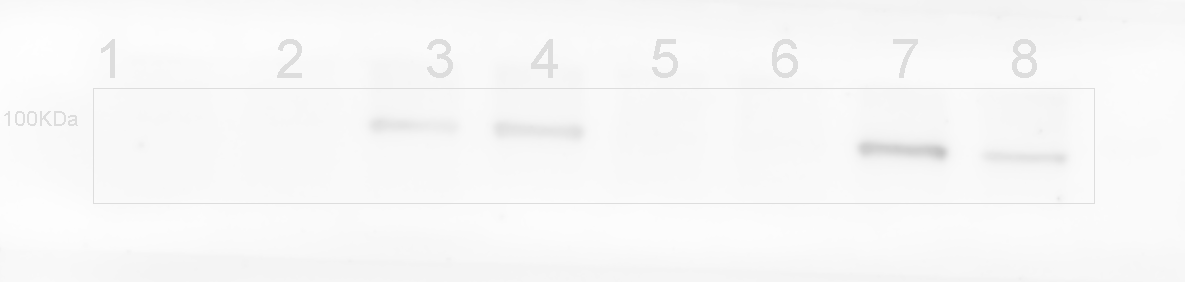

Supplement: Supplementary file 6 — Source data Fig. 4 [file 44319_2025_613_MOESM6_ESM.zip › Figure 4/K/K cleav parp.tif]

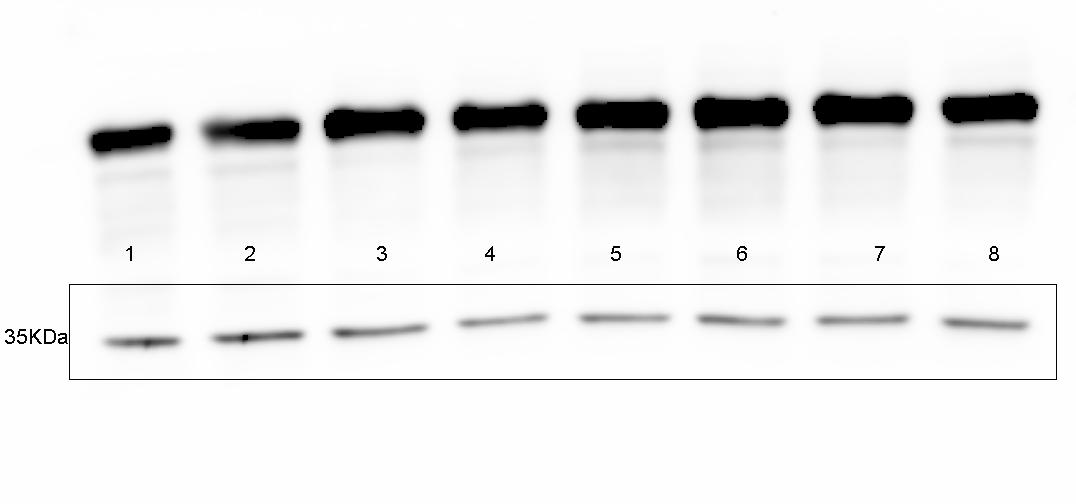

Supplement: Supplementary file 6 — Source data Fig. 4 [file 44319_2025_613_MOESM6_ESM.zip › Figure 4/K/K GAPDH.tif]

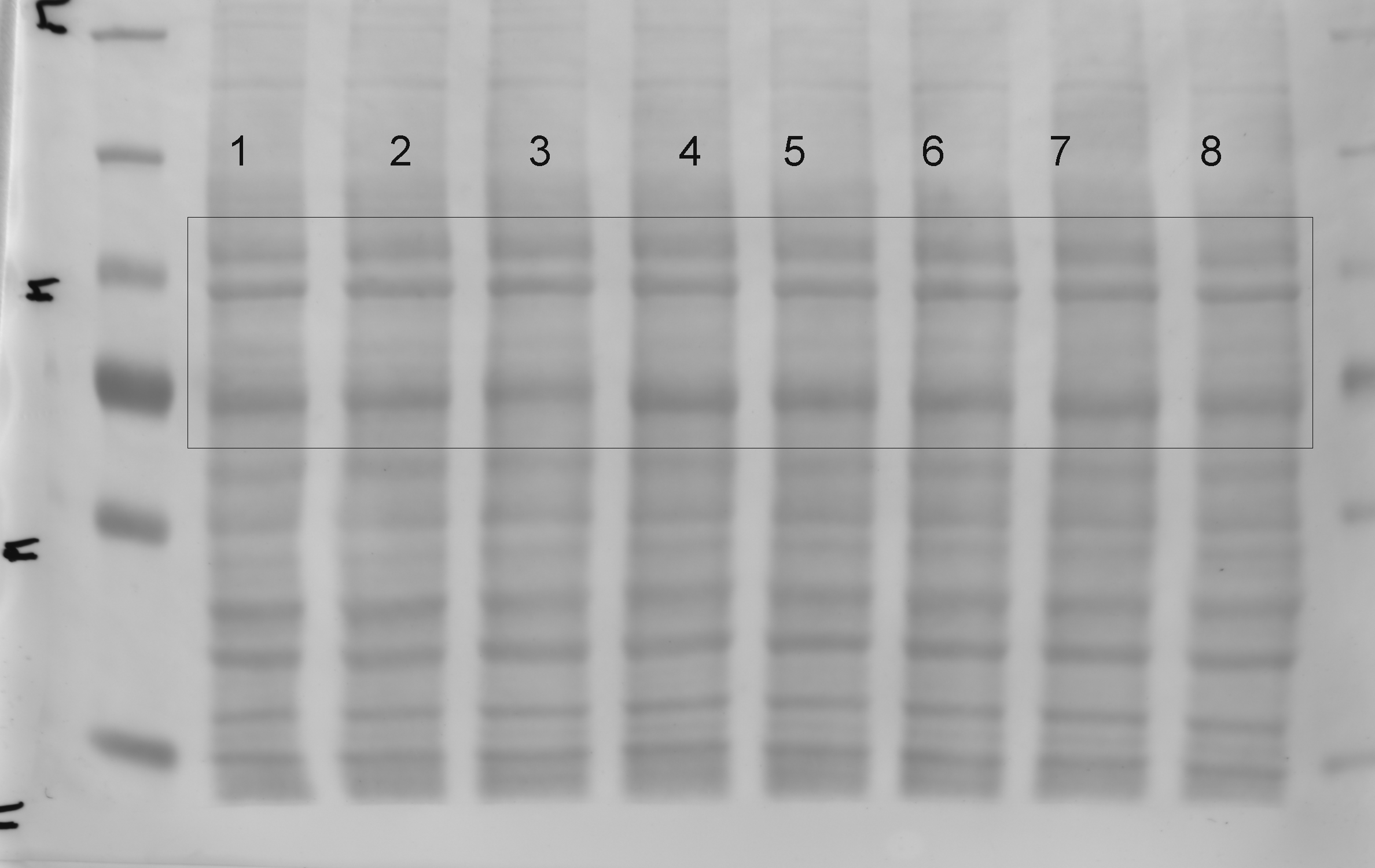

Supplement: Supplementary file 6 — Source data Fig. 4 [file 44319_2025_613_MOESM6_ESM.zip › Figure 4/K/K ponceau.tif]
