# Supplementary material for: Loss of the lysosomal protein CLN3 triggers c-Abl-dependent YAP1 pro-apoptotic signaling
Source: EMBO Rep. 2025 Nov 6;26(24):6096–120. doi: 10.1038/s44319-025-00613-3 (PMC12714701; doi:10.1038/s44319-025-00613-3)
Supplement: Supplementary file 7 — Expanded View Figures [file 44319_2025_613_MOESM7_ESM.pdf]

Expanded View Figures

Figure EV1. Characterization of HEK293T CLN3^{KO} cell line.

(**A**) Schematic representation of the sequence deleted in the CLN3 encoding gene. (**B**) Putative translation sequences showing the premature stop codons. (**C**) Representative immunoblot image and quantification of CLN3 protein levels in HEK293T Wt and CLN3^{KO} cells ($n = 4$). GAPDH was used as loading controls. (**D**) Confocal fluorescence images of HEK293T Wt and CLN3^{KO} cells immunostained for Lamp1 protein and the respective quantification of lysosomal area ($n = 3$). Scale bar 10 and 2 µm in the inset images. (**E**) Quantification of the total number per cell in HEK293T Wt and CLN3^{KO} cells. At least five cells were quantified per independent experiment ($n = 3$). (**F**) Representative immunoblot image and quantification of Lamp1 protein levels in Wt and CLN3^{KO} cells. Ponceau were used as loading controls. (**G**) Confocal fluorescence images of cells HEK293T CLN3^{KO} transfected with GFP or CLN3-GFP and immunostained for Lamp1. Quantification of lysosomal size from at least 50 ($n = 3$). (**H**) Confocal fluorescence images of cells HEK293T parental line transfected with CLN3-GFP and immunostained for Lamp1 ($n = 3$). Scale bar 10 and 2 µm in the inset images. (**I**) Representative fluorescence images of Wt and CLN3^{KO} cells loaded with FITC-dextran (Dx), sensitive to pH, and magic red fluorescence, a substrate of cathepsin B. Scale bar 10 µm. The graphs represent the quantification of FITC-dextran (on the top) and magic red fluorescence (on the bottom), normalized to Alexa 647-dextran, not pH sensitive. At least 15 cells were analysed ($n = 3$). The dashed line represents the cell edge. (**J**) Confocal fluorescence images of cells HEK293T Wt and CLN3^{KO} immunostained for TFEB. The graph represents the TFEB mean intensity in the nuclei of at least 475 cells ($n = 2$). Yellow dashed lines delineate the nuclei. (**K**) Representative immunoblot image and quantification of LC3 protein levels in Wt and CLN3^{KO} cells, untreated and treated with Bafilomycin A1 (BafA1) for 2 h. This blot was also used for the experiment presented in Figure EV3N, and for that reason, the same loading control is used in both panels. Ponceau staining was used as loading controls. Autophagic flux was assessed by the difference between LC3-II levels in BafA1-treated and untreated cells ($n = 4$–5). (**L**) Differentially expressed genes (DEG) between Wt and CLN3^{KO} are represented as a volcano plot: log *p* value adjTtest in the y-axis and log2FC in the x-axis. The transcripts overexpressed in CLN3^{KO} are to the right of the plot, and on the left, the repressed genes. All the results are mean ± SEM. In the violin plots, the orange dots indicate the mean value of each independent experiment. Statistical differences between the two conditions were assessed by unpaired *t*-test (**C**, **D**, **H**, **K**-left graph), Sidak's (**K**, middle graph), or Dunnett's multiple comparisons test (**I**).

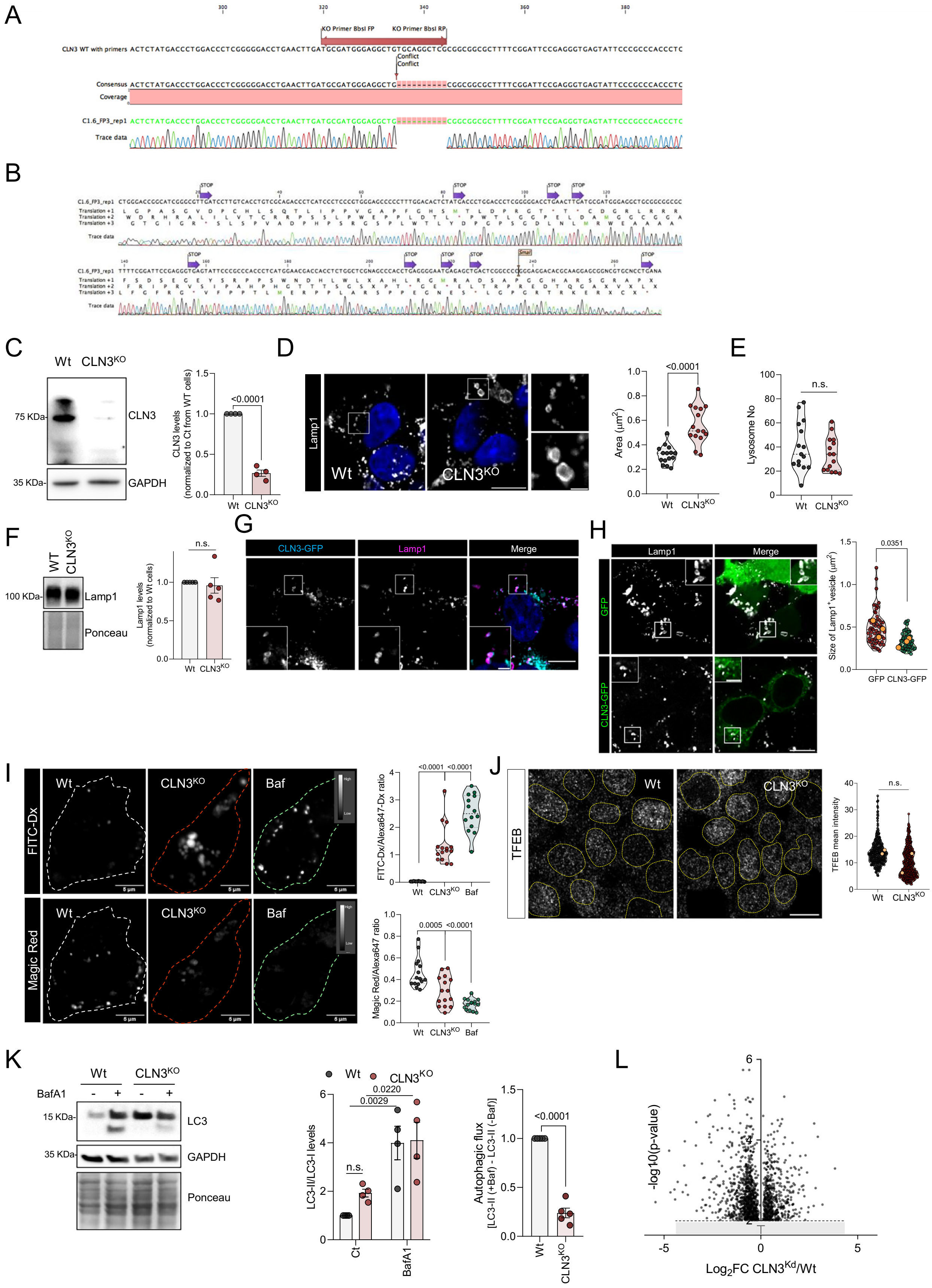
A
B
C
Wt CLN3KO
75 KDa-
CLN3
35 KDa-
GAPDH
CLN3 levels (normalized to Ct from WT cells)
<0.0001
D
Lamp1
Wt
CLN3KO
Area (μm2)
<0.0001
E
Lysosome No
n.s.
F
WT CLN3KO
100 KDa-
Lamp1
Ponceau
Lamp1 levels (normalized to Wt cells)
n.s.
G
CLN3-GFP
Lamp1
Merge
H
Lamp1
Merge
GFP
CLN3-GFP
Size of Lamp1+ vesicle (μm2)
0.0351
GFP CLN3-GFP
I
FITC-Dx
Magic Red
Wt
CLN3KO
Baf
FITC-Dx/Alexa647-Dx ratio
<0.0001 <0.0001
Magic Red/Alexa647 ratio
0.0005 <0.0001
J
TFEB
Wt
CLN3KO
TFEB mean intensity
n.s.
K
Wt CLN3KO
BafA1 - + - +
15 KDa-
LC3
35 KDa-
GAPDH
Ponceau
Wt CLN3KO
LC3-II/LC3-I levels
0.0220
0.0029
n.s.
Ct
BafA1
Autophagic flux [LC3-II (+Baf) - LC3-II (-Baf)]
<0.0001
L
-log10(p-value)
Log2FC CLN3Kd/Wt

 © The Author(s)

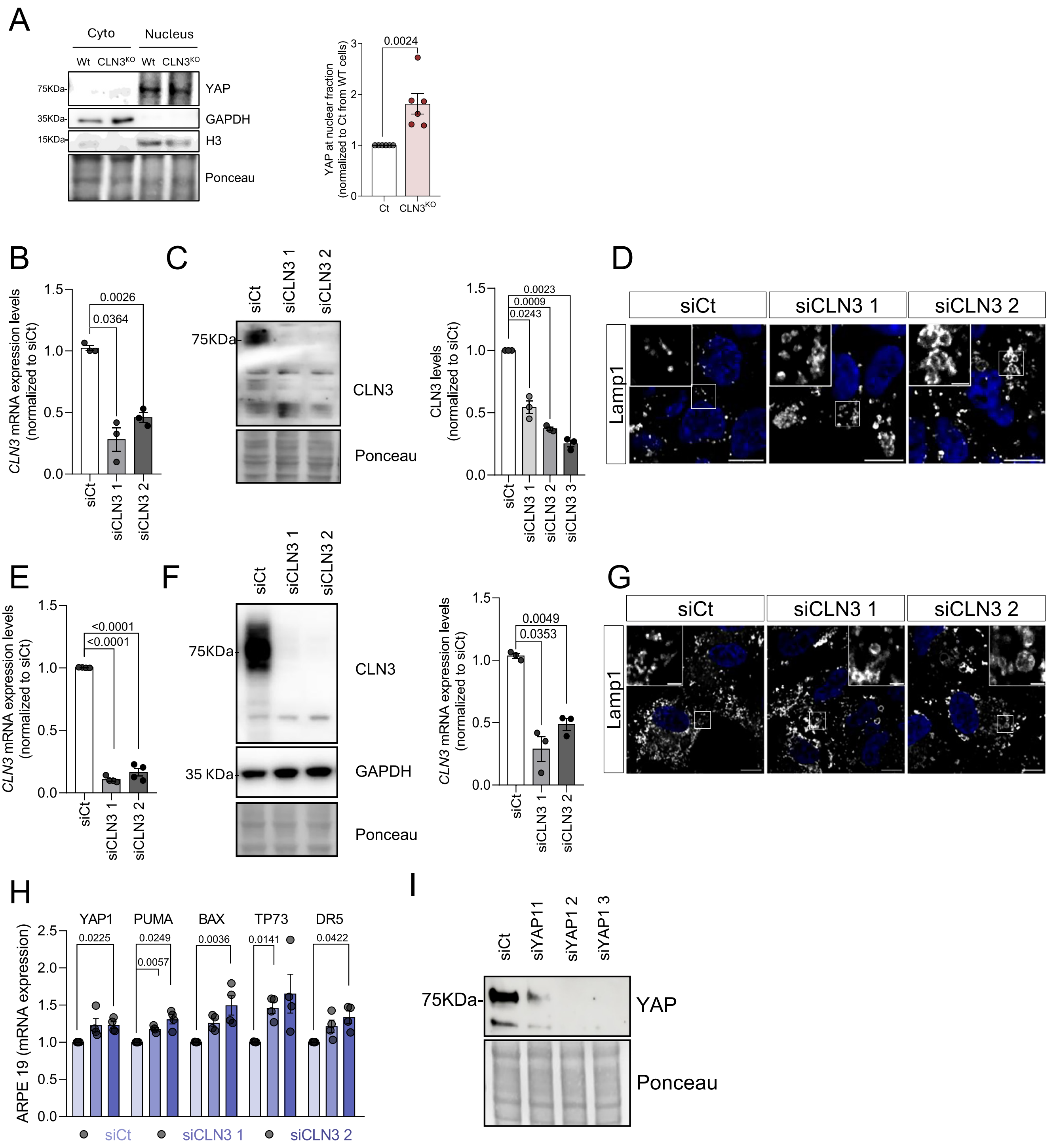
A
Cyto
Nucleus
Wt CLN3KO Wt CLN3KO
75KDa
35KDa
15KDa
YAP
GAPDH
H3
Ponceau
YAP at nuclear fraction (normalized to Ct from WT cells)
0.0024
Ct CLN3KO
B
CLN3 mRNA expression levels (normalized to siCt)
0.0026
0.0364
siCt siCLN3 1 siCLN3 2
C
siCt siCLN3 1 siCLN3 2
75KDa
CLN3
Ponceau
CLN3 levels (normalized to siCt)
0.0023
0.0009
0.0243
siCt siCLN3 1 siCLN3 2 siCLN3 3
D
siCt siCLN3 1 siCLN3 2
Lamp1
E
CLN3 mRNA expression levels (normalized to siCt)
<0.0001
<0.0001
siCt siCLN3 1 siCLN3 2
F
siCt siCLN3 1 siCLN3 2
75KDa
CLN3
35 KDa
GAPDH
Ponceau
CLN3 mRNA expression levels (normalized to siCt)
0.0049
0.0353
siCt siCLN3 1 siCLN3 2
G
siCt siCLN3 1 siCLN3 2
Lamp1
H
YAP1 PUMA BAX TP73 DR5
0.0225 0.0249 0.0057 0.0036 0.0141 0.0422
ARPE 19 (mRNA expression)
siCt siCLN3 1 siCLN3 2
I
siCt siYAP11 siYAP1 2 siYAP1 3
75KDa
YAP
Ponceau

Figure EV2. Characterization of transient modulation of CLN3 and YAP1 levels in HEK293T parental line cell line.

(**A**) Representative immunoblot image and quantification of YAP1 protein levels in the nuclear fraction from Wt and $CLN3^{KO}$ HEK293T cells. Lamin B and GAPDH were used as indicators of nuclear fraction purity ($n = 6$). Ponceau staining was used as loading controls. (**B**) mRNA levels of the CLN3 gene in HEK293T parental line transfected with siCt or two different siRNAs against CLN3 transcript ($n = 3$). (**C**) Representative immunoblot image and quantification of CLN3 protein levels in the conditions described in (**B**) ($n = 3$). Ponceau was used as loading controls. (**D**) Confocal fluorescence images of cells under the conditions described in (**B**). Scale bar 10 μm and 2 μm in the inset images. (**E**) mRNA levels of CLN3 gene in ARPE19 parental line transfected with siCt or two different siRNA against CLN3 transcript ($n = 4$). (**F**) Representative immunoblot image and quantification of CLN3 protein levels in the conditions described in (**E**) ($n = 3$). Ponceau was used as loading controls. (**G**) Confocal fluorescence images of cells under the conditions described in (**E**). Scale bar 10 and 2 μm in the inset images. (**H**) mRNA levels of YAP1 target genes involved in apoptosis in ARPE19 parental line transfected with siCt or two different siRNAs against CLN3 transcript ($n = 4$). (**I**) Representative immunoblot image of YAP1 protein levels from HEK293T parental line transfected with siCt or three different siRNAs against the YAP1 transcript. Ponceau was used as loading controls. All the results are mean ± SEM. Statistical differences between the two conditions were assessed by an unpaired *t*-test (**A**) and Dunnett's multiple comparisons test (**C**, **E**, **F**, **H**).

© The Author(s)

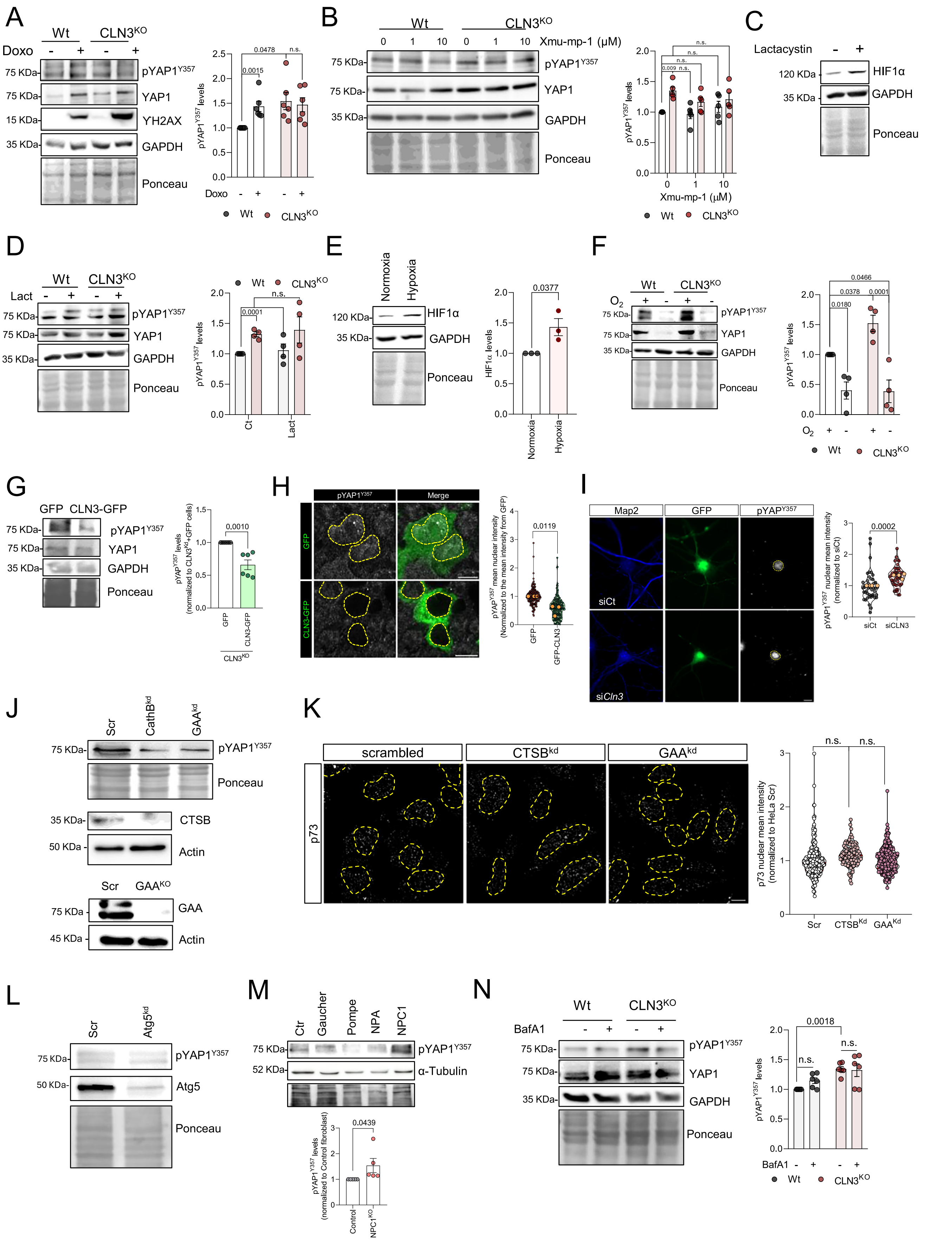

A
Wt
CLN3KO
Doxo
75 KDa
pYAP1Y357
YAP1
15 KDa
YH2AX
35 KDa
GAPDH
Ponceau
pYAP1Y357 levels
0.0478
n.s.
0.0015
Wt
CLN3KO
B
Xmu-mp-1 (μM)
0 1 10 0 1 10
n.s.
0.009
Xmu-mp-1 (μM)
C
Lactacystin
120 KDa
HIF1α
D
Lact
0.0001
Ct
E
Normoxia
Hypoxia
0.0377
HIF1α levels
F
O2
0.0466
0.0378
0.0001
0.0180
G
GFP
CLN3-GFP
0.0010
H
pYAP1Y357
Merge
GFP
GFP-CLN3
0.0119
I
Map2
siCt
siCln3
siCLN3
0.0002
J
Scr
CathBkd
GAAkd
CTSB
50 KDa
Actin
GAAKO
GAA
45 KDa
K
scrambled
CTSBkd
p73
p73 nuclear mean intensity (normalized to HeLa Scr)
L
Atg5kd
Atg5
M
Ctr
Gaucher
Pompe
NPA
NPC1
52 KDa
α-Tubulin
0.0439
Control
NPC1KO
N
BafA1
0.0018

Figure EV3. Restoring CLN3 levels in HEK293T CLN3-KO cells led to a decrease in YAP1 phosphorylation at the Tyr357 residue.

(**A**, **B**) Representative immunoblot image and quantification of pYAP1^{Y357} in (**A**) doxorubicin (Doxo, 25 µM) or (**B**) Xmu-pu-1-treated (1 and 10 µM) or untreated Wt and CLN3^{KO} cells for 24 h ($n = 4$). γH2Ax immunoblot was used as an experimental control to demonstrate increased DNA damage upon doxorubicin treatment. GAPDH and Ponceau were used as loading controls. (**C**) Representative immunoblot image of HIF1α in HEK293T Wt cells after Lactocystin (Lact) treatment for 4 h ($n = 4$). HIF1α, a proteasome substrate, was used as a control of proteasome inhibition efficiency. (**D**) Representative immunoblot image and quantification of pYAP1^{Y357} in Wt and CLN3^{KO} cells in control condition or after Lactocystin (10 µM) treatment in the same conditions as in (**C**) ($n = 3$). (**E**) Representative immunoblot image and quantification of HIF1α in Wt in normoxia and hypoxia. GAPDH and Ponceau were used as loading controls ($n = 4$). (**F**) Representative immunoblot image and quantification of pYAP1^{Y357} (**F**) in Wt and CLN3^{KO} cells in normoxia and hypoxia ($n = 6$). GAPDH and Ponceau were used as loading controls. (**G**) Representative immunoblot image and quantification of pYAP1^{Y357} in CLN3^{KO} cells transfected with pEGFP or CLN3-GFP treated. GAPDH and Ponceau were used as loading controls. (**H**) Confocal fluorescence images and quantification of cells under the conditions described in (**G**) immunostained for pYAP1^{Y357}. Scale bar 10 µm. At least 150 nuclei were analysed ($n = 3$). Nuclei from transfected cells are outlined by the yellow dashed line using Hoechst staining. (**I**) Confocal fluorescence images and quantification of neurons transfected with siCt or siRNA against CLN3 mRNA, and pEGFP (to label transfected cells) and immunostained for pYAP1^{Y357}. Scale bar 10 µm. At least ten nucleus were analysed per independent experiment ($n = 4$). Nuclei are outlined by the yellow dashed line using Hoechst staining. (**J**) Representative immunoblot image of pYAP1^{Y357} in control, Cathepsin B knockdown (CTSB-Kd) and acid alpha-glucosidase knock-down (GAA-Kd) HeLa cells. Protein depletion was also confirmed by CTSB and GAA immunoblotting. Actin, GAPDH or Ponceau were used as loading controls. Scale bar 10 µm. (**K**) Confocal fluorescence images and quantification of cells under the conditions described in (**J**) immunostained for p73. At least a total of 100 nuclei were analysed, 50 in each independent experiment ($n = 2$). Nuclei are outlined by the yellow dashed line using Hoechst staining. Scale bar 10 µm. (**L**) Representative immunoblot image of pYAP1^{Y357} in control and Atg5 knock-down (Atg5-Kd) HeLa cells. (**M**) Representative immunoblot image and quantification of pYAP1^{Y357} (from Nieman–Pick, $n = 5$) in human fibroblast from different lysosomal storage diseases: Gaucher, Nieman–Pick (NPC) and Pompe. Tubulin and ponceau were used as loading controls. (**N**) Representative immunoblot image and quantification of pYAP1^{Y357} in 100 nM of Bafilomycin A1 (BafA1)-treated and untreated Wt and CLN3^{KO} cells for 2 h ($n = 6$). This blot was also used for the experiment presented in Fig. EV1K, and for that reason, the same loading control is used in both panels. GAPDH and Ponceau were used as loading controls. The results are mean ± SEM. In the violin plots, the orange dots indicate the mean value of each independent experiment. Statistical differences between the two conditions were assessed by unpaired *t*-test (**E**, **D**—between Wt and Wt + Doxo, **G**, **H**, **I**, **M**) and Sidak's multiple comparison test (**A**, **B**, **D**, **F**, **K**, **N**).

 © The Author(s)

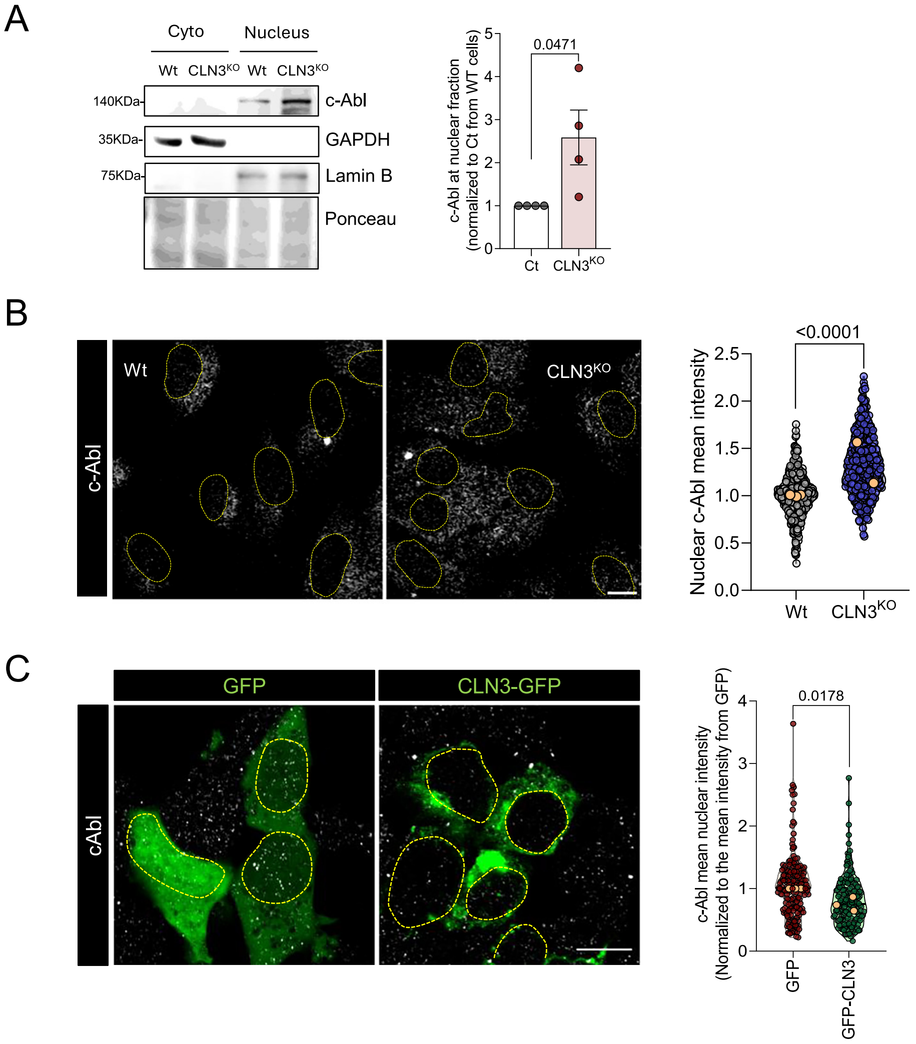

Figure EV4. Restoring CLN3 levels in HEK293T CLN3-KO cells led to a decrease in c-Abl recruitment to the nucleus.

(**A**) Representative immunoblot and quantification of c-Abl in the nuclear fraction of HEK293T Wt and CLN3^{KO} cells ($n = 4$). GAPDH and Lamin B were used as indicators of nuclear fraction purity. (**B**) Confocal fluorescence images and quantification of ARPE19 Wt and CLN3^{KO} cells immunostained for c-Abl. Nuclei are outlined by the yellow dashed line using Hoechst staining. At least 350 nuclei were analysed ($n = 2$). Scale bar 10 µm. (**C**) Confocal fluorescence images and quantification of HEK293T CLN3^{KO} cells transfected with GFP or CLN3-GFP and immunostained for c-Abl. At least 150 nuclei were analysed ($n = 3$). Nuclei from transfected cells are outlined by the yellow dashed line using Hoechst staining. Scale bar 10 µm. The results are mean ± SEM. Scale bar 10 µm. Statistical differences between the two conditions were assessed by an unpaired *t*-test. In the violin plots, the orange dots indicate the mean value of each independent experiment.

© The Author(s)

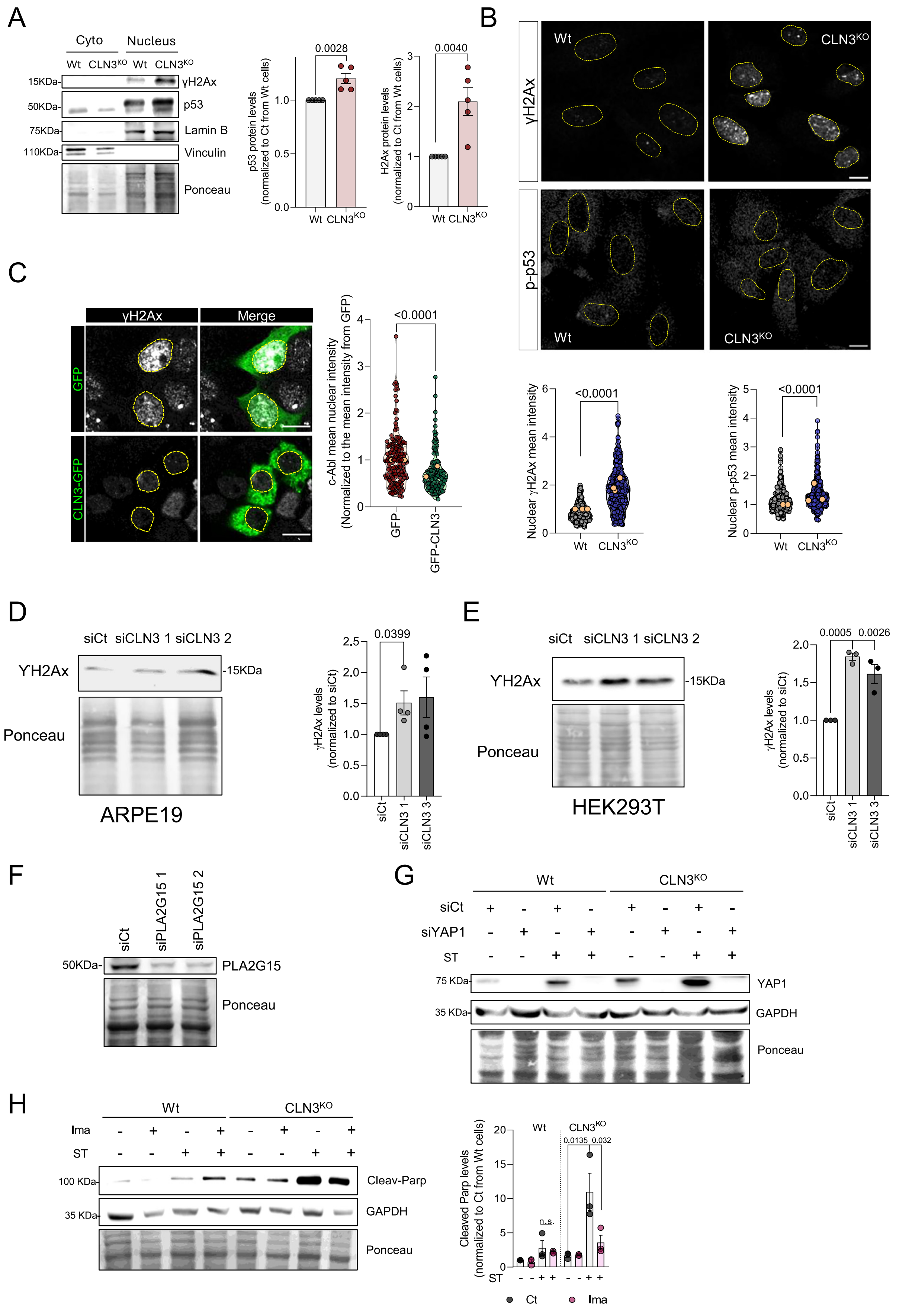
A
Cyto
Nucleus
Wt CLN3KO Wt CLN3KO
15KDa
γH2Ax
50KDa
p53
75KDa
Lamin B
110KDa
Vinculin
Ponceau
0.0028
p53 protein levels (normalized to Ct from Wt cells)
Wt CLN3KO
0.0040
H2Ax protein levels (normalized to Ct from Wt cells)
B
γH2Ax
Wt
CLN3KO
p-p53
<0.0001
Nuclear γH2Ax mean intensity
Nuclear p-p53 mean intensity
C
γH2Ax
Merge
GFP
CLN3-GFP
c-Abl mean nuclear intensity (Normalized to the mean intensity from GFP)
GFP
GFP-CLN3
D
siCt siCLN3 1 siCLN3 2
YH2Ax
-15KDa
Ponceau
ARPE19
0.0399
γH2Ax levels (normalized to siCt)
siCt
siCLN3 1
siCLN3 3
E
HEK293T
0.0005
0.0026
F
siCt
siPLA2G15 1
siPLA2G15 2
50KDa
PLA2G15
G
Wt
CLN3KO
siCt
siYAP1
ST
75 KDa
YAP1
35 KDa
GAPDH
H
Ima
Cleav-Parp
100 KDa
0.0135
0.032
n.s.
Cleaved Parp levels (normalized to Ct from Wt cells)
Ct
Ima

Figure EV5. Loss of CLN3 function induces an increase in DNA damage and cell cycle arrest, possibly by affecting the nuclear lipidome.

(**A**) Representative immunoblot image and quantification of p53 and ϒH2Ax in nuclear extracts from HEK293T parental and CLN3-KO cell line ($n = 5$). Ponceau was used as loading controls and vinculin and Lamin B as control for fraction purity. (**B**) Confocal fluorescence images and quantification of ARPE19 Wt and $CLN3^{KO}$ cells immunostained for ϒH2Ax and p-p53. At least 300 nuclei were analysed ($n = 3$). Nuclei are outlined by the yellow dashed line using Hoechst staining. Scale bar 10 µm. (**C**) Confocal fluorescence images and quantification of HEK293T $CLN3^{KO}$ cells transfected with GFP or CLN3-GFP, treated and immunostained for ϒH2AX. At least 150 nuclei were analysed ($n = 2$). Scale bar 10 µm. (**D**, **E**) Representative immunoblot image of ϒH2Ax in ARPE19 (**D**, $n = 4$) or HEK293T (**E**, $n = 3$) parental cell line transfected with siCt or two siRNAs against CLN3 transcripts. Ponceau was used as loading controls. (**F**) Representative immunoblot image of PLA2G15 in HEK293T parental cell line transfected with siCt or two siRNAs against PLA2G15 transcripts. Ponceau was used as loading controls. (**G**) Representative immunoblot image and quantification of YAP1 of HEK293T (Wt and $CLN3^{KO}$) cells transfected with siCt or siYAP and treated or untreated with staurosporine (ST) treatment (4 h). GAPDH and ponceau were used as loading controls. (**H**) Representative immunoblot image and quantification of cleaved PARP of HEK293T (Wt and $CLN3^{KO}$) cells with or without Imatinib incubation for 24 h, and then treated or untreated with staurosporine (ST) (4 h) ($n = 3$). GAPDH and ponceau were used as loading controls. All the results are mean ± SEM. In the violin plots, the orange dots indicate the mean value of each independent experiment. Statistical differences between the two conditions were assessed by using an unpaired *t*-test (**A**, **B**, **C**, **H**—between $CLN3^{KO}$ cells (Ct and Ima) treated with ST) or Dunnett's multiple comparisons test (**D**, **H**). Scale bar 10 µm.

© The Author(s)
